# Transcriptome analysis of hypoxic cancer cells uncovers intron retention in *EIF2B5* as a mechanism to inhibit translation

**DOI:** 10.1371/journal.pbio.2002623

**Published:** 2017-09-29

**Authors:** Lauren K. Brady, Hejia Wang, Caleb M. Radens, Yue Bi, Milan Radovich, Amit Maity, Cristina Ivan, Mircea Ivan, Yoseph Barash, Constantinos Koumenis

**Affiliations:** 1 Department of Radiation Oncology Perelman School of Medicine, University of Pennsylvania, Philadelphia, Pennsylvania, United States of America; 2 Cellular and Molecular Biology Graduate Group, Perelman School of Medicine, University of Pennsylvania, Philadelphia, United States of America; 3 Department of Biochemistry and Molecular Biophysics, Perelman School of Medicine, University of Pennsylvania, Philadelphia, United States of America; 4 Oncology Center, Zhujiang Hospital of Southern Medical University, Guangzhou, Guangdong, China; 5 Indiana University Health Precision Genomics Program, Indianapolis, Indiana, United States of America; 6 Indiana University Melvin and Bren Simon Cancer Center, Indianapolis, Indiana, United States of America; 7 Center for RNA Interference and Non-coding RNAs, The University of Texas MD Anderson Cancer Center, Houston, Texas, United States of America; 8 Department of Medicine, Indiana University Melvin and Bren Simon Cancer Center, Indianapolis, Indiana, United States of America; 9 Department of Genetics, Perelman School of Medicine, University of Pennsylvania, Philadelphia, United States of America; 10 Department of Computer and Information Science, University of Pennsylvania, Philadelphia, United States of America; University of California at Los Angeles, United States of America

## Abstract

Cells adjust to hypoxic stress within the tumor microenvironment by downregulating energy-consuming processes including translation. To delineate mechanisms of cellular adaptation to hypoxia, we performed RNA-Seq of normoxic and hypoxic head and neck cancer cells. These data revealed a significant down regulation of genes known to regulate RNA processing and splicing. Exon-level analyses classified > 1,000 mRNAs as alternatively spliced under hypoxia and uncovered a unique retained intron (RI) in the master regulator of translation initiation, *EIF2B5*. Notably, this intron was expressed in solid tumors in a stage-dependent manner. We investigated the biological consequence of this RI and demonstrate that its inclusion creates a premature termination codon (PTC), that leads to a 65kDa truncated protein isoform that opposes full-length eIF2Bε to inhibit global translation. Furthermore, expression of 65kDa eIF2Bε led to increased survival of head and neck cancer cells under hypoxia, providing evidence that this isoform enables cells to adapt to conditions of low oxygen. Additional work to uncover *-cis* and *-trans* regulators of *EIF2B5* splicing identified several factors that influence intron retention in *EIF2B5*: a weak splicing potential at the RI, hypoxia-induced expression and binding of the splicing factor SRSF3, and increased binding of total and phospho-Ser2 RNA polymerase II specifically at the intron retained under hypoxia. Altogether, these data reveal differential splicing as a previously uncharacterized mode of translational control under hypoxia and are supported by a model in which hypoxia-induced changes to cotranscriptional processing lead to selective retention of a PTC-containing intron in *EIF2B5*.

## Author summary

Tumor hypoxia is a negative prognostic factor for many solid cancers. Cellular adaptation to hypoxia is largely mediated by widespread changes in gene expression and enables cancer cells to adjust and survive. Recently, alternative splicing has been implicated in this process. To identify biologically impactful hypoxia-responsive isoforms, we took an unbiased approach to deeply sequence RNA of normoxic and hypoxic head and neck cancer cells. This analysis identified >1,000 mRNAs as alternatively spliced under hypoxia, including a significant enrichment of alternatively spliced genes involved in adaptation to hypoxia. Most notably, we discovered a unique retained intron in the translation initiation factor *EIF2B5* that creates a premature termination codon. We show that this retained intron leads to a 65kDa truncated isoform that opposes full-length eIF2Bε to inhibit global translation and enhances survival of head and neck cancer cells under hypoxia. Strikingly, this intron and several additional hypoxia-induced retained introns were overexpressed in solid tumors relative to normal tissues. Mechanistically, we propose that intron retention under hypoxia is influenced by changes to RNA polymerase II (RNAPII) activity at weak 3′ splice sites and carry out experimental validation for the retained intron in *EIF2B5* to investigate intron retention under hypoxia.

## Introduction

Reduced availability of oxygen, or hypoxia, is a major feature of solid tumors that contributes to metastasis and resistance to therapy [1]. Tumor hypoxia occurs due to several physiological factors, such as limited diffusion of oxygen and irregular vascular structure [2]. While oxygen levels can be measured directly in tumors, there is an immediate need to develop noninvasive clinical markers of hypoxic burden in tumors. Molecular imaging markers such as pimonidazole and fluorescence-based compounds [3–5] have been developed and refined to specifically label hypoxic tumors, which allows for specific interrogation of hypoxic gene expression programs within the tumor microenvironment [6]. Hypoxia-mediated changes in expression can be dynamic and robust, impacting pathways critical to tumor development and survival, such as angiogenesis, metabolism, and macromolecular synthesis [7,8]. Consequently, there is a nearly universal negative correlation between the level of hypoxia in tumors and overall survival in patients of many solid cancers, including head and neck squamous cell carcinoma (HNSC) [9]. As such, hypoxia “metagene” expression signatures have been successfully implemented as a surrogate method to classify tumor hypoxia for HNSC and other solid malignancies, including breast and prostate cancer [10,11].

Hypoxic stress influences processing and translation of mRNAs by regulating the levels and activity of diverse factors, including Hypoxia-Inducible transcription Factors (HIFs), small noncoding RNAs and miRNAs, and RNA binding proteins (RBPs) [12–14]. For example, RBPs such as HuR and PTB bind to and regulate the stability and localization of key regulators of hypoxic response such as HIF1α [15,16] and miRNA-199a [17]. Several kinases known to phosphorylate major RBPs and splicing factors are also hypoxia responsive [18]. Consequently, alternative splicing of select target genes of HIF1α has been reported in hypoxic cells [19]. Likewise, expression of noncoding mRNA isoforms are induced under hypoxia in part due to changes in splicing [20]. Several splicing factors, including SF3B1, are up-regulated in a HIF1α-dependent manner under physiological conditions of hypoxia in cardiac myocytes [21]; however, it remains unclear precisely how mRNA splicing is regulated during periods of oxygen deprivation in cancer cells and what the resulting biological implications are. Intriguingly, regulation of splicing is frequently altered in cancer and is affected by the same signaling pathways that are differentially regulated in hypoxic tumor microenvironments [22]. Moreover, many solid cancers affected by hypoxia display widespread alterations in splicing [23].

While splicing of specific genes has been shown to be dependent on the activity of HIF1α, differences in transcription elongation are known to impact regulation of cotranscriptional splicing [24–26]. Thus, we hypothesized that hypoxia-mediated changes to the RNA processing and transcription machinery could lead to extensive differences in mRNA splicing. Hypoxia was identified to induce phosphorylation of the C-terminal domain (CTD) of RNA polymerase II (RNAPII) and was observed to enhance binding of cofactors and increase control of transcriptional activation of HIF target genes [27]. Additional findings support the theory that changes in the activity and rate of transcription elongation play a key role in the maturation and processing of mRNAs under hypoxia. Under hypoxia, there are fewer changes in RNAPII binding near gene promoters but instead an increased accumulation of RNAPII observed along gene bodies [28]. Therefore, to better understand the link between hypoxia-mediated changes to mRNA regulation and to investigate the biological role of alternative splicing in response to hypoxia in cancer, we deeply sequenced mRNA of hypoxic and normoxic HNSC SQ20B cells.

The data led to the identification of more than 1,000 transcripts affected by alternative splicing under hypoxia and revealed 3 types of mRNA splicing as specifically enriched, including an increase in retained introns (RIs) in hypoxia compared to normoxia. Strikingly, for more than 90% of genes in this category, hypoxia increased the occurrence of RIs relative to normoxia, which is a phenomenon also observed in 16 cancer types, including head and neck, colon, breast, and lung cancers [23]. We found evidence of several hypoxia-induced RIs expressed in solid tumor data, indicating that tumor hypoxia contributes to this type of splicing. Most notably, a unique RI in the master regulator of translation initiation, *EIF2B5*, was significantly overexpressed in both head and neck and kidney renal clear cell tumors relative to normal tissues in a stage-dependent manner. Here, we present compelling evidence that hypoxia leads to retention of an intron in *EIF2B5* that creates a phylogenetically conserved premature termination codon (PTC). This alternate transcript results in a truncated protein isoform of eIF2Bε predicted to lack enzymatic guanine exchange factor (GEF) activity. Remarkably, we demonstrate that the resulting truncated isoform of eIF2Bε is induced under hypoxia and provide data to show that this isoform acts in opposition to full-length eIF2Bε to inhibit protein synthesis. Cellular adaptation to hypoxia entails adjusting key metabolic processes, such as translation, to low energy due to reduced availability of oxygen [29]. Control of translation initiation specifically contributes to down-regulation of protein synthesis under hypoxia, which occurs through phosphorylation of eIF2α by hypoxia-mediated induction of the integrated stress response [30]. Here, we discover hypoxia-mediated induction of a dominant-negative isoform of eIF2Bε as a secondary method to inhibit translation and increase survival of head and neck cancer cells in periods of acute or prolonged hypoxia. We further investigated how splicing of *EIF2B5* is controlled and uncovered several factors that contributed to the hypoxia-induced RI, including a weak splice site at the intron:exon junction, hypoxia-induced expression and binding of SRSF3, and an accumulation of RNAPII specifically at the RI. Some of these findings extend to the broader class of genes with RIs under hypoxia, supportive of a mechanism by which changes to RNAPII elongation contributes to retention of introns with weak 3′ splice sites under hypoxia.

## Results

### Hypoxia-mediated changes in transcript-level expression

**Fig 1:**
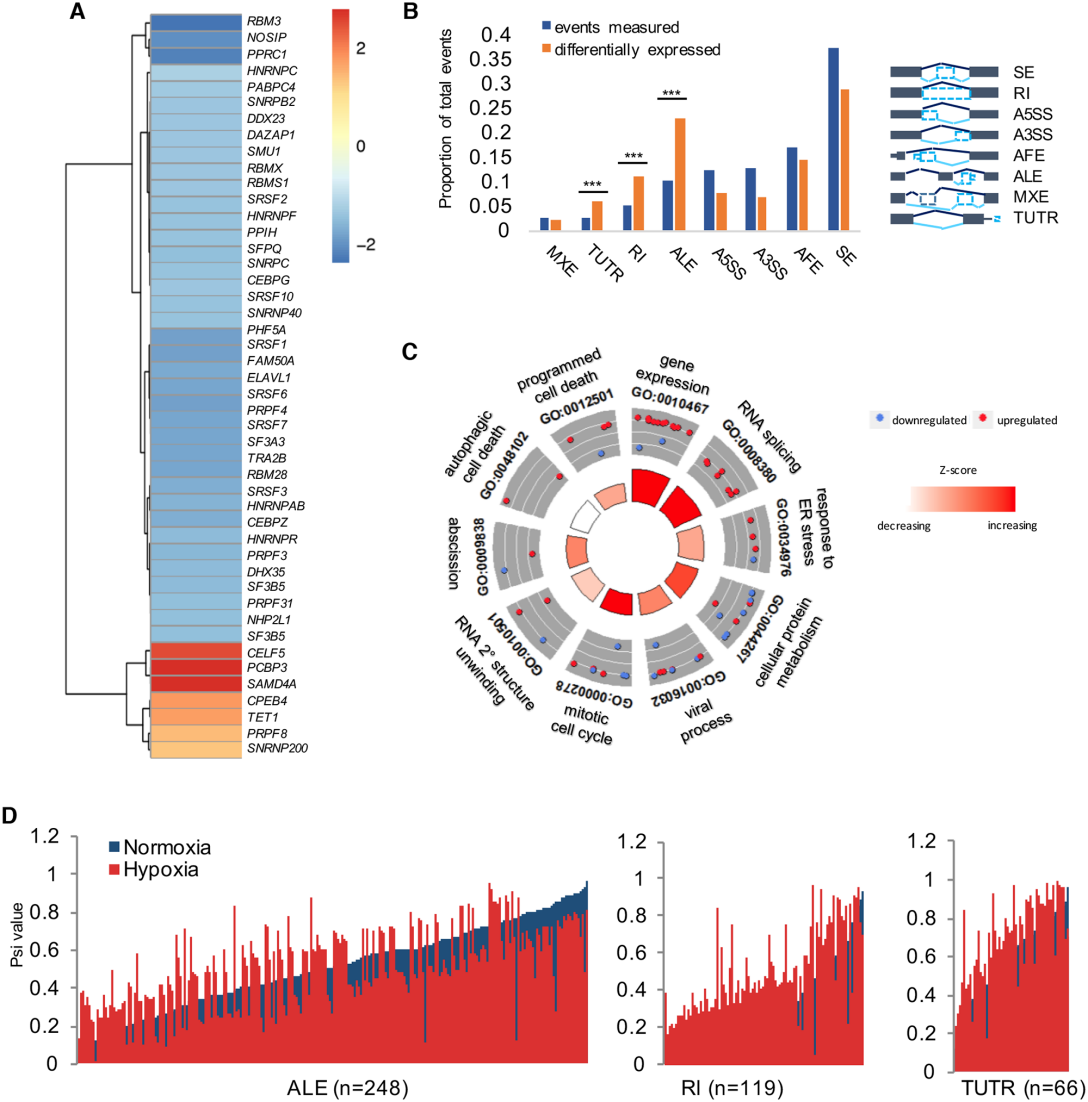
Classification of alternatively spliced mRNAs in hypoxic SQ20B cells. (A) Heatmap of RNA-processing and -splicing factors differentially expressed in hypoxia compared to normoxia (Fold-changes shown, false discovery rate [FDR] < 5%). (B) To the left, plot depicts number of events detected (blue) compared to events with significantly different expression in hypoxic compared to normoxic cells (red) (Bayes Factor ≥ 20, ΔѰ > 10%; Abbreviations: A3SS, alternative 3′ splice site; A5SS, alternative 5′ splice site; AFE, alternative first exon; ALE, alternative last exon; MXE, mutually exclusive exon; RI, retained intron; SE, skipped exon; TUTR, tandem 3′ untranslated region). Specific enrichment for changes in 3 event types are starred: ALE, RI, and TUTR (***P < 0.001, 2-sample test for equality of proportions). To right of graph, exon models of the types of splicing assessed by MISO analysis. (C) Gene ontology figure representing functional enrichment for hypoxia-induced changes in ALE, RI, and TUTR categories. (D) Percent spliced in (Psi) values plotted with hypoxia samples (red) overlaid against corresponding normoxic Psi values (blue). All supporting data used in the generation of this figure are included in S1 Data.

The transcriptomes of normoxic and hypoxic SQ20B cells (maintained in 0.5% O_2_ for 16 h) were compared to identify expression differences at an individual mRNA transcript level. The RefSeq hg19 reference comprised of 46,017 transcript models was used for annotation. Of the 24,812 transcripts expressed in SQ20B cells, we detected 3,114 that significantly changed expression in hypoxia compared to normoxia (*P* < 0.05, false discovery rate [FDR] < 5%, Fragments Per Kilobase of transcript per Million mapped reads [FPKM] ≥ 0.5). In total, 1,519 transcripts representing 1,473 genes were induced and 1,595 transcripts expressed from 1,563 genes were repressed (FDR < 5%). Pathway-based analysis identified “cell adhesion,” “response to hypoxia,” and “metabolism” among the most enriched categories for hypoxia-induced transcripts (*P* < 0.01, DAVID GO [31]). Induction of select HIF1α target genes was validated by quantitative PCR (qPCR) (S1A Fig). Repressed genes were involved in regulating processing, stability, and translation of RNAs, with “ribosome biogenesis,” “nucleosome organization,” and “RNA splicing” as highly significant ontology groups (*P* < 0.01). This included core regulators of alternative splicing, such as the major splicing factor *SF1*, several serine–arginine splicing factors (*SRSF1*, *SRSF3*, and *SRSF7)*, *SF3* genes, and many translation initiation factors, including *EIF2B* family members, *EIF5*, and *EIF6*. Notably, a closer examination of genes involved in regulation of mRNA transcription, translation, and processing revealed that the clear majority of these genes were repressed under hypoxia (Fig 1A).

Variation in expression of individual transcripts of the same gene by hypoxia could be masked when analyzing expression changes at the total gene level; therefore, we used levels of individual isoforms to classify genes as hypoxia-responsive (defined as a gene with 1 or more individual transcripts detected as significantly induced or repressed, *P* < 0.05, FDR < 5%). This method uncovered 50 additional genes that may not yet be reported as hypoxia-regulated, including genes known to mediate protein localization (*SEC24B*, *ARL17A*, *PLEKHA8*, *MLPH*, *STXBP5*, *MON2*, and *EXOC1*), Mitogen-activated protein kinase kinase signaling pathway (*MAP4K3*, *DUSP22*, and *MAP3K13*), and key regulators of RNA metabolism (*ZNF519*, *JDP2*, *CTBP2*, *ZNF248*, *RBM5*, *NFAT5*, *CREB3L2*, *SMARCA1* and *ZFHX3*). Consistent with the finding that transcript-level changes comprise an additional layer of hypoxia-regulated expression changes, only approximately 3% of genes that expressed multiple isoforms in SQ20B cells have been reported to be transcriptionally controlled by HIF binding at hypoxia-responsive elements (HRE) [32], suggesting an HRE-independent mode of regulation. This is not surprising, as regulation of transcription and mRNA splicing are observed to be distinct processes that impact different subsets of genes [33].

Next, we focused on those genes that expressed more than 1 transcript in SQ20B cells to assess changes in patterns of isoform expression. Altogether, of the 5,418 genes identified to express >1 transcript, 937 of these genes showed hypoxia-induced changes in expression of 1,015 transcripts (*P* < 0.05, FDR < 5%). Most these genes contained a single hypoxia-responsive isoform; only 8% of genes that expressed multiple transcripts showed more than 1 isoform that significantly changed expression in hypoxia. The isoform-level changes in expression were validated for select genes that displayed differential expression of isoforms predicted to have different biological functions (S1B Fig). Hypoxia selectively induced the *MXI1* isoform, which codes for the shortest protein isoform that would lack 46 amino acids at the N-terminus compared to full-length isoforms. This difference was determined to alter the ability of the truncated protein to antagonize N-Myc activity and impact cell proliferation [34]. Likewise, the isoform of *NDRG1* that exhibited the strongest induction arises from an alternative transcription start site, which leads to 66 fewer amino acids and exclusion of a proteolytic cleavage site that remains in the other two isoforms of *NDRG1*. Transcripts of 2 additional genes, *NEK6* and *FAM86C1*, were validated as being selectively repressed under hypoxic conditions (S1C Fig).

### Functional classification of changes in alternative splicing

**Fig 2:**
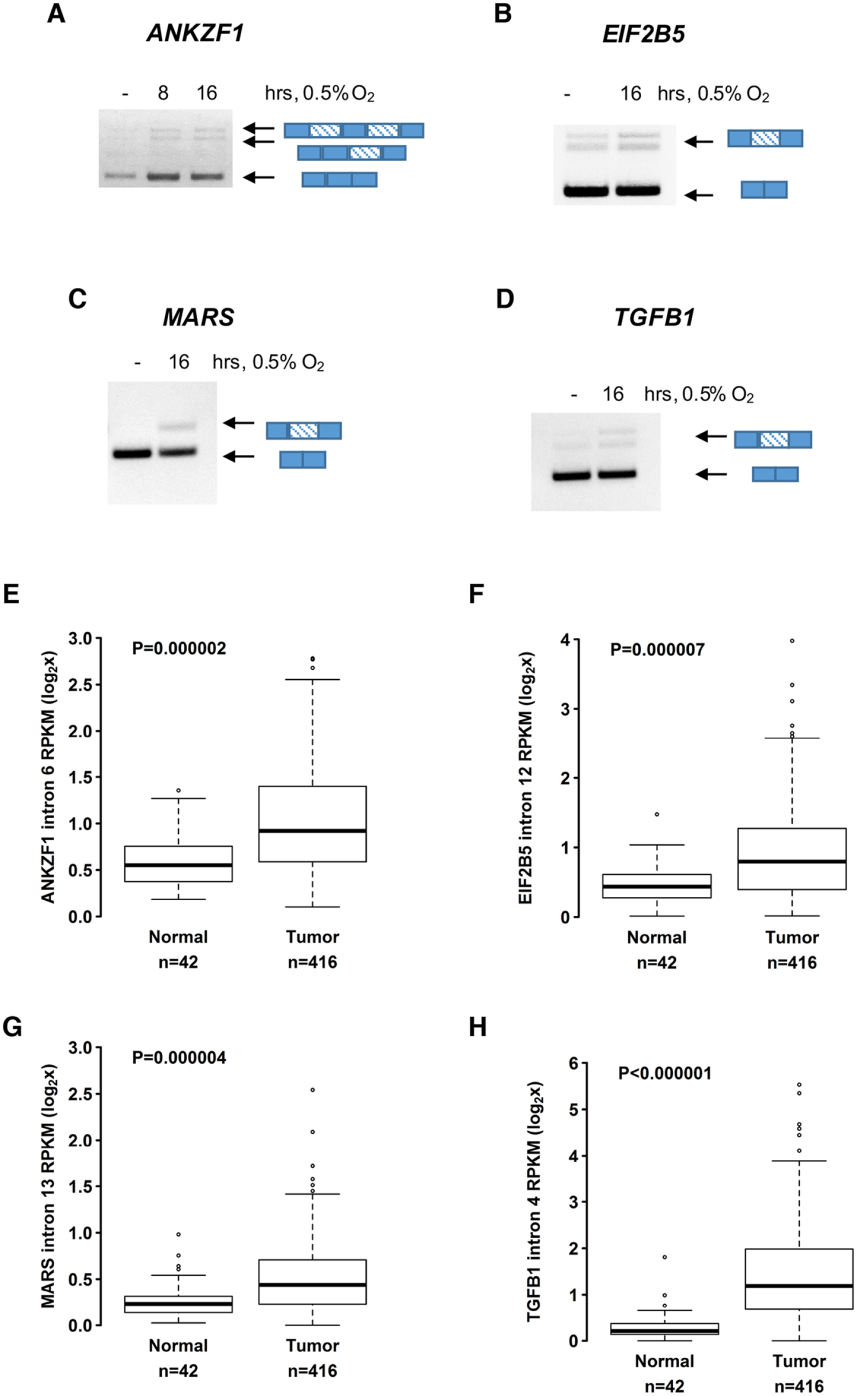
Hypoxia-induced retained introns (RIs) for several genes are confirmed by PCR and expression analysis of head and neck tumors. For each panel, PCR validation of intron retention events using cDNA prepared from oligo-dT–selected mRNA treated with DNAse enzymes is shown. Genes include (A) *ANKZF1*, (B) *EIF2B5*, (C) *MARS*, and (D) *TGFB1*. Diagrams beside gel images of PCR products depict gene locus models with exons as solid blue and introns as striped rectangles. For each gene, expression analysis for HNSC tumor and matched normal tissue data is shown below—(E) *ANKZF1*, (F) *EIF2B5*, (G) *MARS*, and (H) *TGFB1*. Data used in the generation of this figure are included in S1 Data.

We reasoned that hypoxia-responsive isoforms predicted to carry out different functions than isoforms expressed in normoxia would be the most biologically impactful changes that warranted further study. Therefore, we used the program MISO [35] to carry out an additional exon-level approach to identify changes in gene structure based on 8 annotated categories of alternative splicing. Hypoxia led to a change in expression for 1,103 alternatively spliced loci representing 819 unique genes (Fig 1B, ΔPsi > 10%, Bayes Factor > 20). Notably, there was a significant comparative enrichment for hypoxia-induced changes in 3 specific event types: expression of alternate last exons (ALEs), RIs, and tandem 3′ UTRs (TUTR) (Fig 1B). For the genes in these 3 splicing categories, gene ontology revealed processes central to hypoxic adaptation as significantly enriched, including “cellular protein metabolism,” “programmed cell death,” and “gene expression” (Fig 1C, DAVID GO, *P* < 0.05, S1 Data). Remarkably, nearly 90% of the genes in the RI category displayed increased retention of introns in hypoxic compared to normoxic cells (Fig 1D). Among these genes was *ANKZF1*, a gene implicated in mitochondrial and endoplasmic reticulum-associated protein degradation [36,37], the translation initiation factor *EIF2B5*, *TGFB1*, and the metionyl-TRNA synthetase, *MARS*. The RIs in these genes were validated by PCR using primers spanning the intron junction and cDNA prepared from oligo-dT–selected mRNA (Fig 2A–2D, S2 Fig).

To confirm expression of these hypoxia-induced introns in additional datasets and determine if they were expressed in patient samples, we interrogated The Cancer Genome Atlas (TCGA) and analyzed expression solid tumors known to be affected by hypoxic fractions [38–40]. This analysis validated increased expression of the hypoxia-induced RIs for *ANKZF1*, *EIF2B5*, *MARS*, and *TGFB1* in HNSC tumors relative to matched normal tissues (Fig 2E–2H). The extended analysis of additional cancer types confirmed significantly increased expression of these hypoxia-induced RIs for 2 types of renal carcinoma, as well as lung, liver, and prostate cancers (S3 Fig).

**Fig 3:**
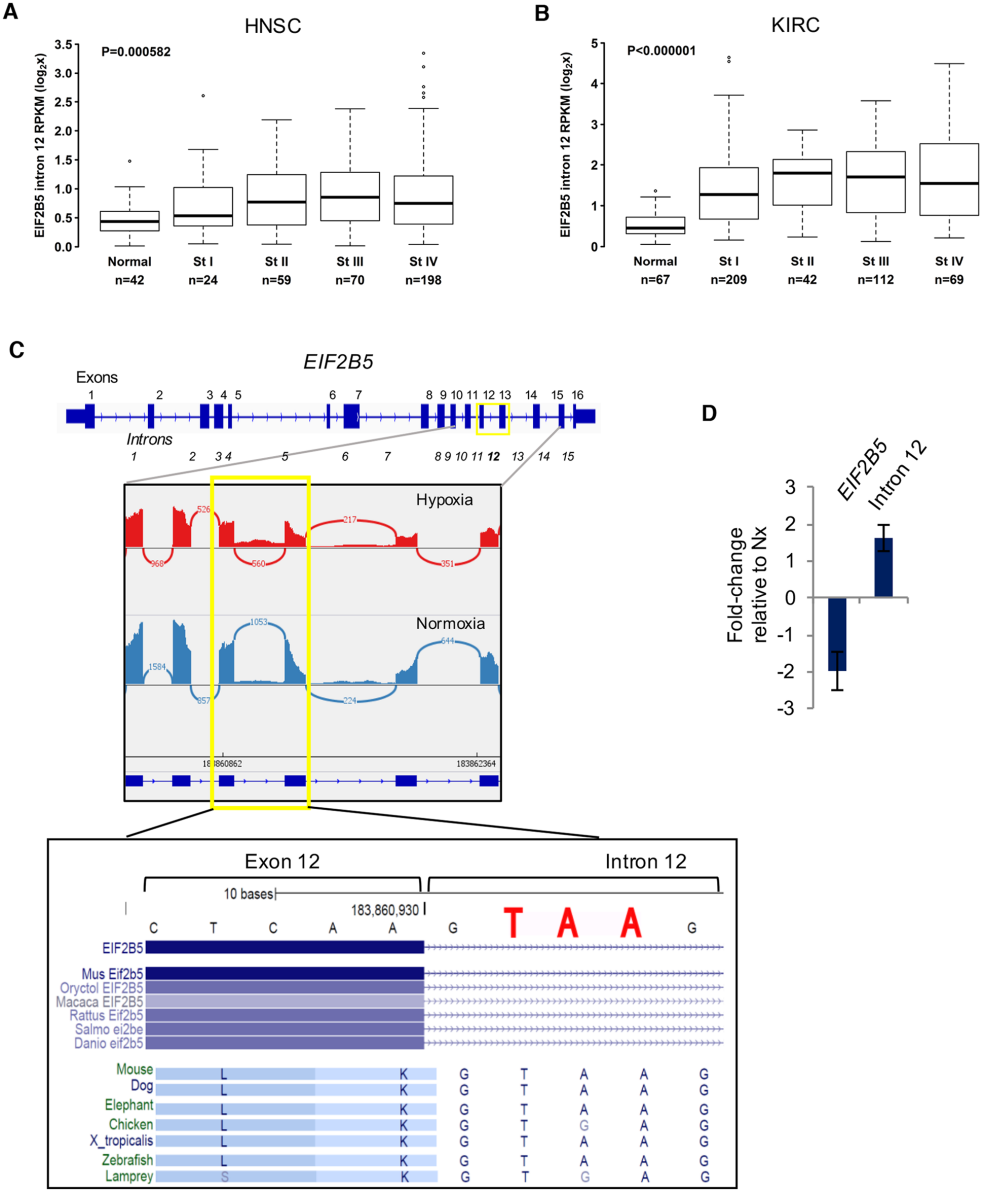
The clinical and biological relevance of a hypoxia-induced retained intron in *EIF2B5*. (A) Expression of *EIF2B5* intron 12 was measured in head and neck squamous cell carcinoma (HNSC) tumors compared to matched normal tissues and reported according to disease stage. (B) Expression of *EIF2B5* intron 12 was measured in kidney renal clear cell carcinoma (KIRC) tumors compared to matched normal tissues and reported according to disease stage. (C) Upper: Gene model of *EIF2B5*, with sashimi plot of normalized RNA-Seq reads for representative matched hypoxia and normoxia samples (plot created using Integrative Genome Viewer). Lower: UCSC Genome Browser Vertebrate Conservation track reveals a conserved stop codon (TAA or TGA) that remains in frame upon retention of intron 12. (D) Real-time quantitative PCR performed with isoform-specific primers. Data are presented as average of 3 independently conducted experiments, with error bars reported as SEM. All supporting data used to create this figure can be found in S1 Data.

Intron 12 of *EIF2B5* showed a strong stage-dependent increase in expression for both head and neck and kidney renal clear cell carcinomas (KIRC) (Fig 3A and 3B). This trend was even more apparent with HNSC patients in late-stage disease, with some individuals exhibiting nearly 8-fold increased expression of intron 12 compared to controls (Fig 3A). These data suggest hypoxia-induced retention of *EIF2B5* intron 12 may result in meaningful biological effects and encouraged us to examine the functional impact of this RI.

The RI in *EIF2B5* stood out as the strongest candidate for functional studies for several additional reasons: (a) hypoxia led to a >40% increase in retention of intron 12 in a background of an overall 2-fold decrease in total expression of *EIF2B5* (ΔѰ = 0.44, Bayes Factor > 20); (b) the retention occurred specifically at a single locus of *EIF2B5*; and (c) retention of intron 12 creates a PTC that remains in frame with the coding sequence (Fig 3C). The genomic locus around the PTC is highly conserved, even among lower organisms. Intriguingly, this stop codon is part of the 5′ splice site consensus sequence “GURAGU,” where URA can be either UAA or UGA (both of which would create a stop codon). This suggests a strong evolutionary pressure to preserve an early termination precisely at this location, where inclusion of the PTC may be influenced through regulation of splice site choice.

Increased expression of *EIF2B5*_intron12 was further confirmed by qPCR using intron-specific primers in a reaction with cDNA prepared from oligo-dT–selected mRNA (Fig 3D). Additionally, a deeper analysis of changes in splicing of *EIF2B5* was carried out to closely examine inclusion of intron 12 and to validate the occurrence of this RI using another method; the software package MAJIQ [41] was used to assess local splicing variation in *EIF2B5* for annotated and de novo splice events. A hypoxia-induced increase in expression of intron 12 was confirmed using this approach (S4 Fig), but additional sites of local splicing variation did not show significant (>20%) hypoxia-influenced changes.

### Retention of intron 12 in *EIF2B5* leads to a truncated protein isoform

**Fig 4:**
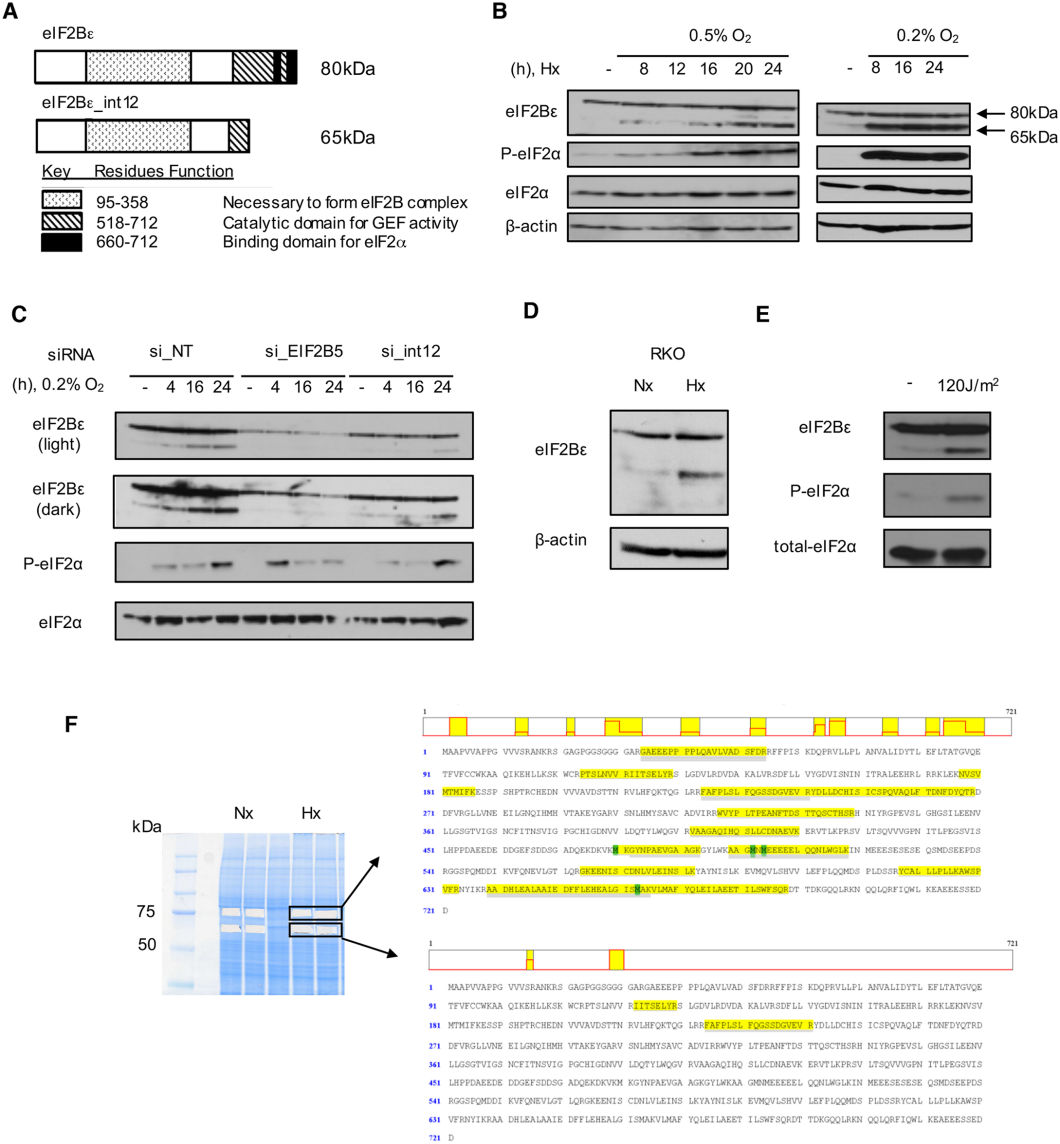
Retention of intron 12 leads to a 65kDa isoform of eIF2Bε. (A) Protein model of observed isoforms of eIF2Bε. (B) Immunoblot shows induction of a 65kDa isoform of eIF2Bε in SQ20B cells maintained for various periods in 0.5% or 0.2% O_2_. (C) Knock-down of eIF2Bε with siRNA targeting the whole gene or intron 12 reduces expression of the 65kDa isoform in normoxic and hypoxic (0.2% 0_2_) SQ20B cells. Abbreviation: NT, non-targeting control siRNA. (D) Hypoxia (16h, 0.5% O_2_) in a colon cancer cell line, RKO, leads to induction of 65kDa eIF2Bε. (E) Immunoblot of SQ20B cells collected 4h after exposure to ultraviolet (UV). (F) Protein sequence of eIF2Bε with peptides identified from liquid chromatography tandem mass spectrometry (LC-MS/MS) sequencing highlighted in yellow (shown to right). Analysis was carried out from gel-purified bands (shown left) corresponding to approximately 80kDa (top) and 65kDa (lower), which contained peptides of full-length eIF2Bε and a truncated isoform of eIF2Bε, respectively.

Computational analyses approximate up to 20%–35% of alternatively spliced transcripts could contain PTCs and become targets of nonsense-mediated decay (NMD) [42,43]; however, in cases where NMD is inhibited, transcripts can be stabilized and subsequently translated into truncated proteins [44]. Moreover, NMD surveillance typically recognizes stop codons as premature if the stop occurs more than 50 nucleotides upstream of a splice junction [45]. Due to the unusual nature of this intron retention event and the role of hypoxia in suppressing NMD in an eIF2α phosphorylation-dependent manner [46], we predicted that this isoform would not be subject to NMD but would rather be translated into a truncated protein (Fig 4A). Consistent with this hypothesis, the MAJIQ splicing analysis of *EIF2B5* revealed a 40%–50% decrease in expression of remaining exons following intron 12 (S4 Fig). These data support the notion that transcripts that retain intron 12 and the subsequent PTC would undergo read-through of intron 12 into intron 13, resulting in a reading frame for a truncated protein variant. Indeed, we observed induction of a 65kDa protein isoform of eIF2Bε under various conditions of hypoxia consistent with the predicted PTC inserted upon retention of intron 12 (Fig 4B). Induction of phospho-eIF2α was used as a marker for hypoxia in these experiments (Fig 4B). To further test whether this 65kDa protein detected in the immunoblot was indeed a truncated isoform of eIF2Bε, we used small interfering RNA (siRNA) to specifically target the entire *EIF2B5* gene or intron 12 alone. Using this approach, we observed a substantial reduction in the levels of the 65kDa isoform under both conditions (Fig 4C, S5A Fig). We additionally observed hypoxia-induced expression of 65kDa eIF2Bε in the colorectal cancer cell line RKO (Fig 4D), demonstrating that this is not a cell-line specific event. Moreover, ultraviolet (UV) radiation (Fig 4E), but not thapsigargin-induced endoplasmic reticulum (ER) stress (S5B Fig), also led to induction of the truncated eIF2Bε, suggesting that expression of this isoform is induced by specific cell stresses. Furthermore, whole-cell lysates were isolated from hypoxic SQ20B cells, and proteins migrating at 80kDa and 65kDa were subjected to liquid chromatography tandem mass spectrometry (LC-MS/MS). Peptides corresponding to eIF2Bε were detected in both the 80kDa and 65kDa size analytes. The peptides corresponding to the 65kDa-sized isoform of eIF2Bε were located near the N-terminus or middle of the eIF2Bε sequence, consistent with a C-terminal truncation (Fig 4F). To additionally rule out that expression of the band migrating at 65kDa is a degradation product or may occur due to proteolytic cleavage, we used a plasmid to express a version of eIF2Bε with a C-terminal FLAG-tag in SQ20B cells and exposed these cells to UV radiation. If the tagged eIF2Bε were to undergo proteolytic cleavage, we predicted to observe a 15kDa band reactive to FLAG-tag antibody in addition to the 65kDa band reactive against eIF2Bε antibody in UV-treated cells. This experiment did not show evidence of the 15kDa tagged proteolytic cleavage product (S5C Fig). Finally, we carried out an experiment to test for the involvement of NMD in leading to the expression of truncated eIF2Bε. Although NMD is known to be inhibited in conditions of oxygen deprivation [46], NMD and alternative splicing are coupled processes, which can lead to alternative PTC-containing transcripts that ultimately become targets of NMD [47]. To rule out any major contribution of NMD in leading to expression of 65kDa eIF2Bε, we used siRNA to knock down expression of UPF1. UPF1 is a necessary component of the SURF complex, which is required for NMD [48,49]. This experiment failed to produce an increase in expression of the truncated protein (S5D Fig), further supporting the notion that this isoform occurs due to alternative splicing.

### Search for *-cis* and *-trans* regulators of alternative splicing of *EIF2B5*

**Fig 5:**
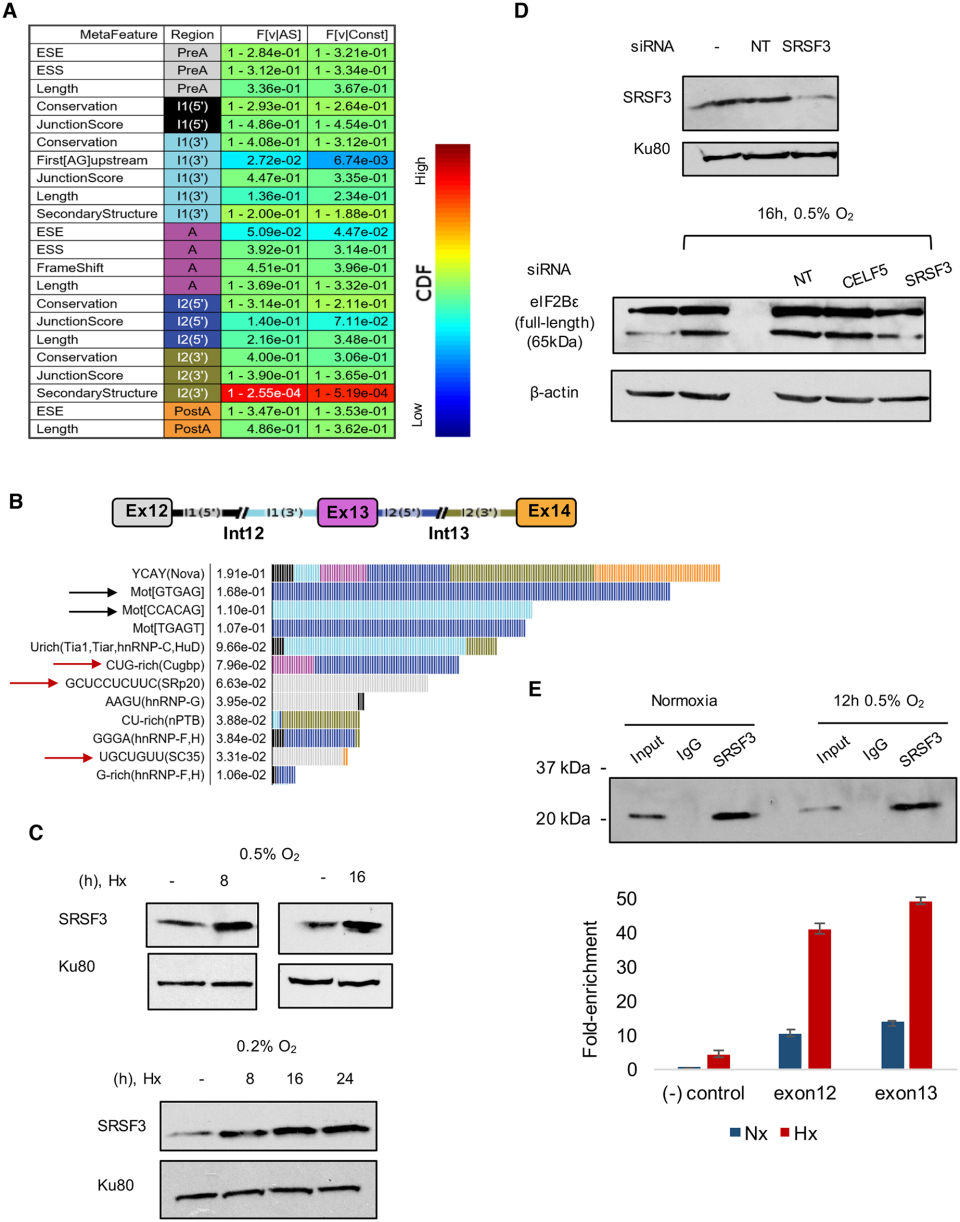
Analysis of RNA binding factor motifs and regulatory sequence features at the *EIF2B5* intron12 locus. (A) A nonmotif analysis of other sequence features influencing splicing of *EIF2B5* exons 12–14. These were data generated using AVISPA [50]. (B) Splicing factor motifs determined to have the largest effects on regulation of the *EIF2B5* exons 12–14 are shown, with color-coded gene map above and predicted regulatory sites shown below with feature effect value. Black rows highlight a predicted weak splice site before exon 13 and an alternate GTGAG splice site after exon 13. Red arrows signify splicing factors observed as hypoxia-responsive in RNA-Seq data. (C) Immunoblot of SQ20B lysates to assess expression of SRSF3 protein under hypoxia. Nx = normoxia, Hx = 16 h 0.5% O_2_ hypoxia. (D) Upper: Immunoblot analysis of knock-down efficiency of SRSF3 in SQ20B cells. Lower: Immunoblot of lysates collected from SQ20B cells treated with 50 nM siRNA under 16 h 0.5% O_2_ hypoxia. (E) Upper: Immunoblot results from immunoprecipitation of SQ20B lysate with SRSF3 antibody in normoxic and hypoxic cells. Rabbit IgG was used as a control. Lower: Reverse transcription quantitative PCR (RT-qPCR) analysis of RNA isolated from the immunoprecipitation with SRSF3 using primers for negative (-) control (a region of GAPDH predicted to contain no binding of SRSF3) and primers for the 2 exons flanking *EIF2B5* intron12 predicted to have SRSF3 binding. Additional data used in the generation of this figure are included in S1 Data.

To identify potential regulators mediating retention of intron 12 in *EIF2B5*, we utilized the AVISPA tool [50] to carry out a splicing-relevant sequence feature analysis of the locus encompassing *EIF2B5* exons 12–14. The nonmotif analysis revealed a relatively short distance to the nearest AG dinucleotide upstream of exon 13, as well as relatively unstructured RNA immediately downstream of exon 13 (Fig 5A). Both features indicate weakened splicing potential of exon 13 [51,52]. The motif analysis revealed sequence motifs, such as potential RBP binding motifs, which could be important for regulating splicing of the locus (Fig 5B). Notably, these sequence features were not identified in a search of additional loci within *EIF2B5*. As a control, we used AVISPA to predict the occurrence of alternative splicing in 4 additional exon triplets within *EIF2B5* (exons 6–8, 7–9, 8–10, and 9–11). These loci were not predicted to be alternatively spliced. Altogether, these results suggest that alternative splicing is significantly more likely to occur at the intron 12 locus compared to the control loci tested.

The motif analysis also identified potential *trans* regulators with binding sites within this locus predicted to regulate splicing of this region, including *NOVA*, *HNRNPC*, and members of the CUGBP Elav-Like Family (CELF), as well as Ser/Arg-rich splicing factor 3 (*SRP20*, AKA *SRSF3*), *HNRNPG*, *NPTB*, *HNRNPF*, and Ser/Arg-rich splicing factor 2 (*SC35*, AKA *SRSF2*) (Fig 5B). Interestingly, several of the splicing factors predicted to have the greatest impact on regulation of this locus also changed expression under hypoxia, including *SRSF2*, *SRSF3*, *HNRNPC*, *HNRNPF*, and *RBMX* (AKA *HNRNPG*), which were repressed at the mRNA level, and *CELF5* (a member of the CELF family), which was induced (Fig 1A, FC ≥ |1.2|, FDR < 5%). Upon closer examination of these splicing factors, we focused in on SRSF3 and CELF5 as prime candidates to test as regulators of *EIF2B5* splicing, due to a large number of binding sites near the intron12:exon13 junction (S6A Fig) and the fact that these factors showed >2-fold changes in mRNA expression under hypoxia.

We next assayed for changes in protein expression of these splicing factors in hypoxic cells and observed a reproducible hypoxia-induced increase in SRSF3 protein (Fig 5C, S6B Fig) and a modest decrease in expression of CELF5. To test for their requirement in the splicing of *EIF2B5* and production of the resulting 65kDa protein isoform, we used siRNA to knock-down expression of these factors and assayed for changes in expression of 65kDa eIF2Bε. Upon knockdown of SRSF3, we saw a concurrent disappearance in the expression of the 65kDa isoform of eIF2Bε in conditions of normoxia or hypoxia (Fig 5D), while siRNAs against other RBPs, such as CELF5, did not display the same effect (S6C and S6D Fig). RNA immunoprecipitation assays were next carried out to confirm a direct interaction between SRSF3 protein and *EIF2B5* RNA. The data showed enrichment of SRSF3 at both exons flanking *EIF2B5*_intron12 (Fig 5E), validating the predicted binding sites observed in other systems (S6A Fig). Furthermore, binding of SRSF3 was increased in hypoxic cells relative to normoxic cells, providing additional support for the role of SRSF3 in regulating hypoxia-induced retention of intron 12.

### Hypoxia-mediated changes in regulation of RNAPII may influence retention of *EIF2B5* intron 12

**Fig 6:**
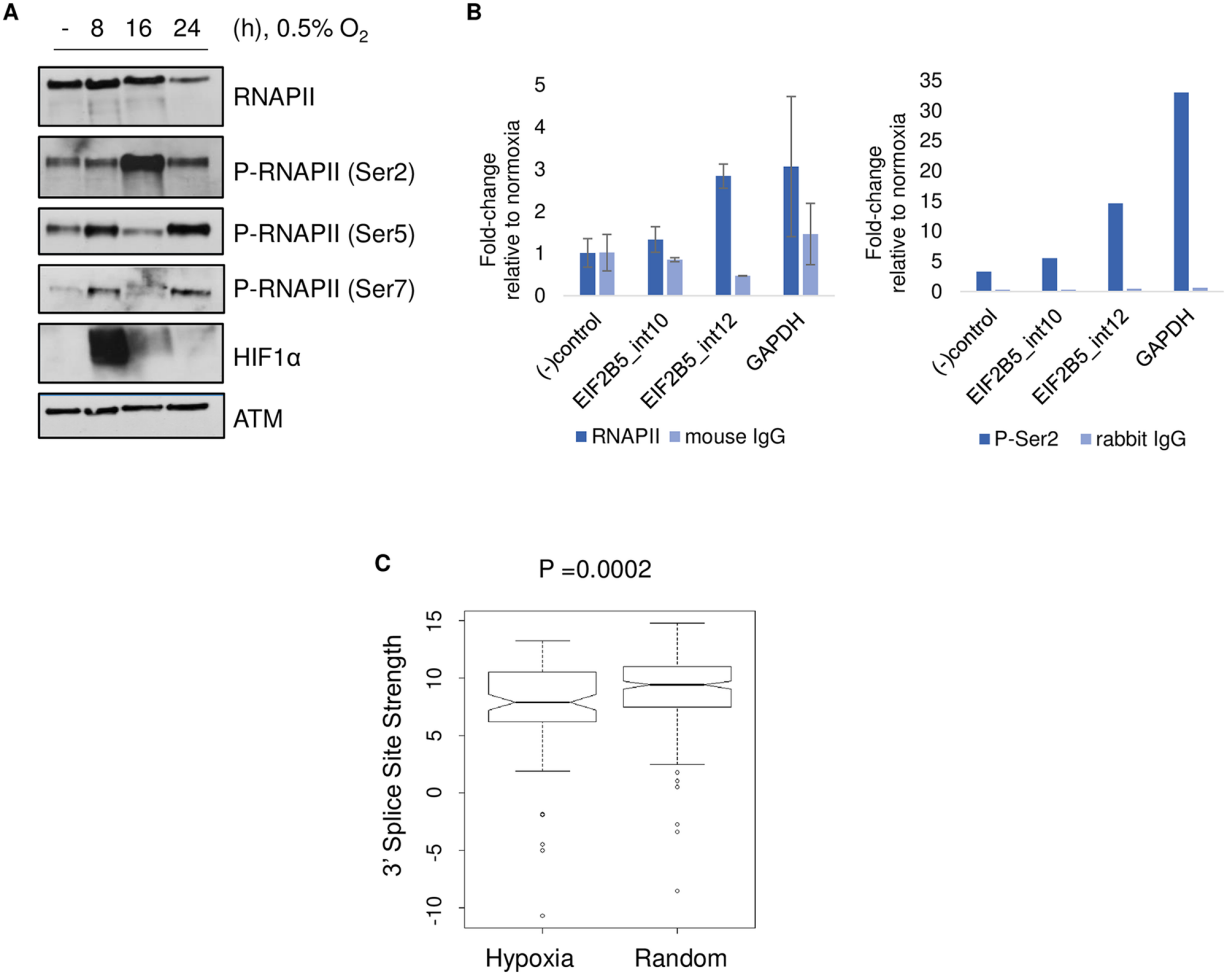
Detection of hypoxia-mediated changes in phosphorylation and binding of RNA polymerase II (RNAPII). (A) Immunoblot of phosphorylated forms of RNAPII in nuclear lysates of SQ20B cells. Expression of ATM (Ataxia Telangiectasia Mutated Serine/Threonine kinase) was used as a loading control. (B) Chromatin immunoprecipitation followed by quantitative PCR (qPCR) to determine abundance of total RNAPII or P-Ser2 RNAPII at EIF2B5 intron 12, an upstream negative control intron 10, a negative control region of GAPDH ((-) control), and a RNAPII-positive control region of GAPDH. For (B), total RNAPII data represent average of *n* = 3 independently conducted experiments (error bars = SEM) and P-Ser2 data are shown as an average of *n* = 2 independently conducted experiments. (C) Analysis of 3′ splice sites carried out using a First Model Markov method to determine maximum entropy scores, reported as 3′ splice site strength [56]. Hypoxia group = 101 unique 3′ splice sites of introns retained under hypoxia; Random group = 252 hg19 3′ splice sites. Additional data used to create this figure are included in S1 Data.

In addition to the influence of sequence elements, mRNA splicing is a co-transcriptionally regulated process which is impacted by coordination of RNAPII. Phosphorylation of the CTD of RNAPII is known to impact the rate of transcriptional elongation, pausing, and the relative rate of splicing [53,54]. Therefore, we assayed for changes in phosphorylation of the CTD of the largest subunit of RNAPII in HNSC cells. Surprisingly, we saw a large (>10-fold) and reproducible increase in levels of phosphorylation at serine 2 residues and a concomitant decrease in phosphorylation of serine 5 and 7 residues of the CTD in hypoxic cells compared to normoxic controls (Fig 6A, S7 Fig). Interestingly, accumulation of the phospho-Ser2 form of RNAPII has been associated with RIs compared to constitutively spliced introns [55]. To assay for changes in binding of total and phospho-Ser2 RNAPII, we next used chromatin immunoprecipitation followed by qPCR. We observed a significant hypoxia-induced enrichment in both forms of RNAPII at intron 12 but did not detect the same enhanced binding at another nearby intron of *EIF2B5* that did not undergo intron retention under hypoxia (Fig 6B).

In strong support of these data, cells expressing mutant forms of RNA polymerase with slower elongation rates led to extensive changes in mRNA splicing, including specific retention of *EIF2B5* intron12 [57]. Moreover, 31 of 100 genes we identified to be affected by intron retention in hypoxia were classified as “rate-sensitive” and displayed altered expression in the RNA polymerase elongation mutants [57], suggesting that hypoxia-mediated changes in elongation of RNAPII likely influence splicing of additional loci. Those genes alternatively spliced in conditions of slow elongating RNAPII generally exhibited weaker 3′ splice sites compared to loci insensitive to changes in elongation rate [57], so we next carried out an analysis of splice site strength for genes affected by intron retention under hypoxia. This analysis detected significantly weaker 3′ splice sites for genes with changes in RIs under hypoxia (score = 7.6) compared to a control set of splice sites not affected by hypoxia (score = 8.9) (Fig 6C, *P* = 0.00289). This included intron 12 of *EIF2B5*, where the 3′ splice site strength score was 1.1 points lower compared to the 3′ splice site strength of the control set.

### The 65kDa isoform of eIF2Bε acts in opposition to full-length eIF2Bε to inhibit protein synthesis

**Fig 7:**
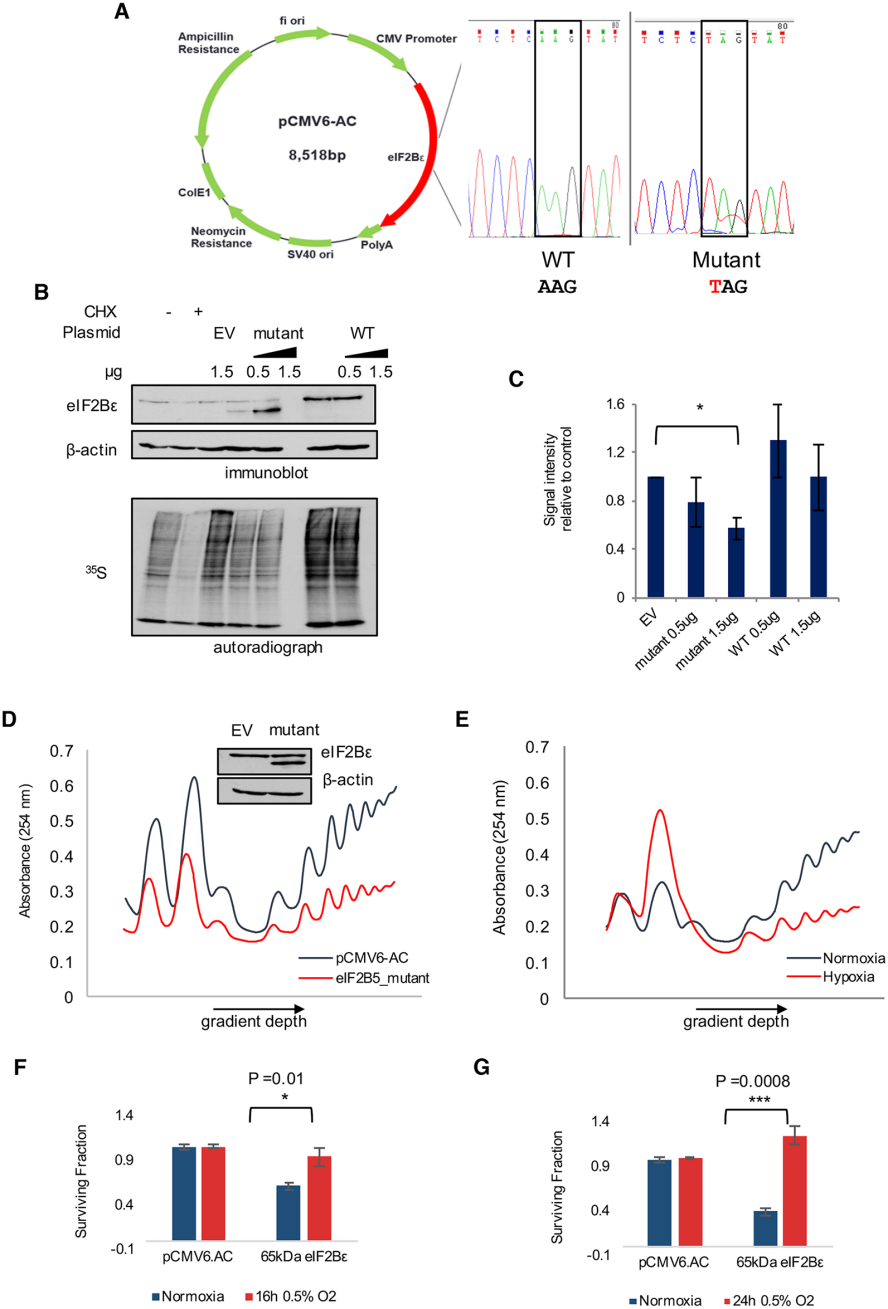
Expression of a 65kDa isoform of eIF2Bε leads to a global decrease in protein synthesis. (A) Site-directed mutagenesis was used to introduce a stop codon into *EIF2B5* (within 1 codon of where intron retention introduces an early stop). (B) Expression of full-length (wild-type [WT]) or mutated eIF2Bε (mutant) was performed for 24 h in SQ20B cells (upper), followed by pulse-labeling with ^35^S methionine/cysteine (lower) to measure changes in protein synthesis compared to expression of an empty vector pCMV6-AC (EV). Cells treated with cyclohexamide (CHX) were used as a control for inhibition of translation initiation. (C) Image quantification of total ^35^S signal from autoradiographs of 4 independently conducted experiments. Data collected from *n* = 4 independently conducted experiments, *P < 0.01 (Student *t* test). (D) Polysome profile depicts SQ20B cells expressing the 65kDa isoform of eIF2Bε for 36 h compared to cells expressing control pCMV6-AC, with supporting immunoblot shown above. (E) Polysome profiling of hypoxic versus normoxic SQ20B cells. (F) Clonogenic assay of SQ20B cells expressing control plasmid (pCMV6.AC) or 65kDa eIF2Bε in normal oxygen or 0.5% O_2_ for 16 h. Analysis of 3 biological replicates quantified to right (*P* value reported for Student *t* test). (G) Clonogenic assay of SQ20B cells expressing control plasmid (pCMV6.AC) or 65kDa eIF2Bε in normal oxygen or 0.5% O_2_ for 24 h. Analysis of 3 biological replicates quantified to right (*P* value reported for Student *t* test). Additional data used to create this figure are included in S1 Data.

Next, we tested whether the expression of 65kDa eIF2Bε has an impact on the biological function of the endogenous, full-length eIF2Bε protein. The truncated protein isoform created from retention of intron 12 is predicted to lack 2 critical domains that occur in the C-terminus (Fig 4A): a GEF domain and a region required for interaction with eIF2α [58]. Thus, we hypothesized that the 65kDa isoform of eIF2Bε would inhibit translation and lead to reduced protein synthesis. To test this, we constructed a plasmid expressing the truncated 65kDa isoform using site-directed mutagenesis to insert a stop codon within 3 nucleotides of where retention of intron 12 results in a PTC (Fig 7A). Expression of this mutated version of eIF2Bε under normoxic conditions resulted in the appearance of a 65kDa protein isoform consistent with the size of the endogenous protein that is induced under hypoxia (Fig 7B).

To analyze the impact of the truncated isoform on protein synthesis, we used pulse-labeling of ^35^S methionine/cysteine in cells expressing 65kDa eIF2Bε, full-length eIF2Bε, or empty vector. Proteins isolated from the ^35^S methionine/cysteine-labeled cells were resolved on SDS-PAGE, after which the gels were dried and exposed to autoradiograph film to detect signal intensities as a measure of total protein synthesis. There was a pronounced and reproducible decrease (approximately 30%) in total protein synthesis in cells expressing 65kDa eIF2Bε compared to empty vector, while expression of full-length eIF2Bε did not have the same effect (Fig 7B and 7C).

Translation levels were assessed relative to cells expressing empty vector as a control in order to best isolate the effects of the truncated isoform from those of endogenous full-length eIF2Bε. Effects of inducing full-length eIF2Bε were not used as a control because full-length eIF2Bε remains stably expressed under hypoxic conditions and is not induced. In addition, expression of eIF2B has been shown to destabilize ternary complex formation under conditions where eIF2 is phosphorylated [59].

**Fig 8:**
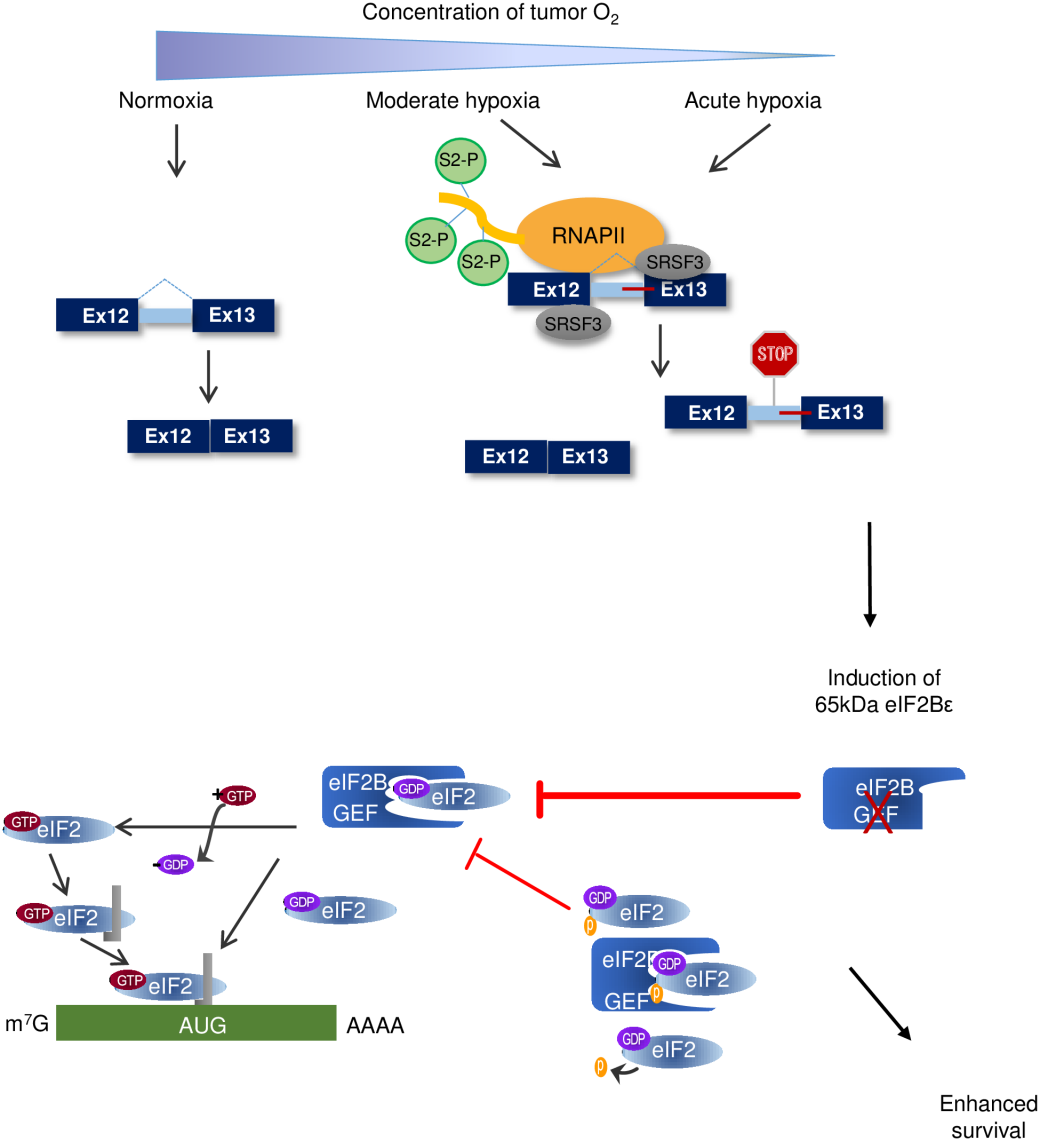
Model of hypoxia-induced intron retention in *EIF2B5* as a mechanism to reduce translation and enhance survival in head and neck cancer cells during periods of prolonged or acute hypoxia. Upper: Under acute or prolonged hypoxia, increased phosphorylation of Ser2-RNAPII accumulates specifically at *EIF2B5* intron 12. Binding of SRSF3 is increased under hypoxia at this locus, which contains a weak splice site and alternate downstream splice site. Altogether, oxygen deprivation leads to an accumulation of intron12-containing *EIF2B5* transcripts, which results in a truncated reading frame due to insertion of a premature termination codon (PTC). Lower: Retention of intron 12 under hypoxia results in a 65kDa isoform of eIF2Bε. This isoform lacks the functional guanine exchange factor (GEF) domain and acts opposite to the full-length isoform to inhibit translation during periods of prolonged hypoxia, which ultimately confers a survival advantage to SQ20B cells under hypoxia.

To further verify the effects observed in the ^35^S assay, we carried out polysome profiling as an additional measure of protein synthesis. The data confirmed a decrease in the total polysome profiles of cells expressing 65kDa eIF2Bε compared to control cells (Fig 7D, S8A Fig). Moreover, this decrease in translation was comparable to the decrease in translation observed in hypoxic conditions (Fig 7E). Consistent with these data, we observed an increase in adenosine triphosphate:adenosine monophosphate (ATP:AMP) ratio in cells expressing 65kDa eIF2Bε relative to cells expressing empty vector or the full-length isoform (S8B Fig). Finally, because down-regulation of translation is a known mechanism by which tumor cells overcome hypoxic stress [60], we predicted that expression of 65kDa eIF2Bε and the resulting repression of translation may promote survival of hypoxic cells. To test this, clonogenic assays were performed to measure survival and proliferation. The surviving fraction of cells expressing 65kDa eIF2Bε decreased under normoxic conditions but was significantly higher when cells were grown in 0.5% O_2_ conditions for either 16 h or 24 h (Fig 7F and 7G, S8C and S8D Fig, *P* < 0.01, Student *t* test). Collectively, these data establish a role for the 65kDa isoform of eIF2Bε in reducing protein synthesis in head and neck cancer cells to adapt to conditions of hypoxia (Fig 8). Mechanistically, we provide strong evidence that expression of this isoform is influenced by differential binding of SRSF3 at the locus of *EIF2B5* intron12, a weak splice site coupled with an alternate splice site, and increased binding of total and P-Ser2 RNAPII (Fig 8).

## Discussion

This study has validated alternative splicing as a major, additional layer of complexity to the gene expression response to hypoxia. Previous work had described hypoxia-mediated changes in splicing, including in several known HIF-target genes [19,61] and further identified splicing as a means to induce expression of noncoding RNAs under hypoxia [20]. Here, we expand upon the current understanding of RNA processing under hypoxia and demonstrate that decreased oxygen in cancer cells leads to extensive changes in splicing, including a striking increase in retention of over 100 introns affecting a significant number of genes with key functional roles in cellular adaptation to hypoxia. As such, a major focus of our study became to investigate the biological significance of hypoxia-induced intron retention in the master regulator of translation initiation, *EIF2B5*.

Specific retention of intron 12 in *EIF2B5* led to a previously undescribed 65kDa isoform of eIF2Bε that decreases overall protein synthesis in head and neck cancer cells. Full-length eIF2Bε is a necessary component of the eIF2B complex, containing both the active GEF domain and a region for association with eIF2α [62]; this complex binds eIF2α and exchanges guanosine diphosphate (GDP) for guanosine triphosphate (GTP) to initiate translation. During hypoxia, eIF2α is phosphorylated, and translation initiation is inhibited and was demonstrated to be critical for cell survival under extreme hypoxia [60]. However, this phosphorylation is not long-lasting and can be removed by GADD34 [29,63]. Moreover, in cells stably expressing an unphosphorylatable form of eIF2α with an S51A mutation, translation initiation resumed under hypoxia, but only to approximately 75% of the level observed in control cells [60]. These data suggested the existence of additional mechanisms to sustain reduced protein synthesis in conditions of low oxygen. Thus, we propose that, during periods of acute or prolonged hypoxia, intron retention in *EIF2B5* leads to expression of a truncated dominant-negative isoform to further inhibit protein synthesis in cancer cells (Fig 8). Expression levels of 65kDa eIF2Bε are consistently up-regulated in hypoxia, with the greatest induction of this isoform observed at stringent (0.2% O_2_) and prolonged (≥ 16 h) hypoxic conditions (Fig 4B). Moreover, SQ20B cells expressing 65kDa eIF2Bε demonstrated an increase in ATP:AMP ratio and increased clonogenicity in conditions of low oxygen (S8 Fig). Altogether, these data establish a biological role for the 65kDa isoform of eIF2Bε in promoting adaptation to hypoxic stress in cancer cells.

The question remains as to whether induction of this isoform is a cause or effect of tumorigenesis; additional animal studies are necessary to evaluate its potential to influence tumor formation and will be a focus of future work. Intriguingly, altered regulation of eIF2B GEF activity and translational control has been observed in transformed cells relative to primary cells [64]. In conditions that activate the unfolded protein response (UPR), transformed cells displayed increased levels of GDP exchange from eIF2 relative to normal human cell lines, despite comparable levels of phospho-eIF2α [64]. These data provide an additional line of evidence that cancer cells in conditions of stress may require mechanisms outside of phospho-eIF2α to control eIF2B GEF activity and overall levels of protein synthesis.

Our findings led to the first reported case of splicing as a previously uncharacterized mode of translational control under conditions of hypoxia. The mechanism behind hypoxia-induced alternative splicing is influenced by several factors, including both *-cis* RNA sequence determinants and oxygen-sensitive *-trans* regulators. We used retention of intron 12 in *EIF2B5* as a starting point to investigate regulation of intron retention in hypoxic cells. Several unique aspects of intron 12 may lead to its retention in *EIF2B5*, such as a weak 3′ splice site and a weakened splicing potential in this region of *EIF2B5* influenced by a relatively short distance to the nearest AG dinucleotide upstream of exon 13, as well as the relatively unstructured RNA immediately downstream of exon 13. Intron length and GC content near the splice site can also influence splice site choice [65,66]. Alternatively, RIs often exhibit lower GC content compared to the immediate upstream and downstream exons [66]. Interestingly, the GC content of *EIF2B5* intron 12 was 6% lower than that of the adjacent downstream exon 13. This was the intron:exon junction where our AVISPA analysis detected a relatively weaker splice site and a possible “GT” alternate splice site downstream in intron 13. The unique properties of this locus may promote specific retention of intron 12 compared to other introns under hypoxia.

The influence of hypoxia-responsive *-trans* regulators on splicing of *EIF2B5* was also substantial. Our data support the theory that hypoxia-mediated changes in expression of splicing factors and regulation of transcription elongation contribute to splice site choice. We observed down-regulation of many genes that regulate splicing and RNA processing, including some that were predicted to bind at the *EIF2B5* intron 12 locus. There is evidence that global down-regulation of splicing factors and RBPs can promote intron retention in a regulated manner during other physiological responses or processes, such as hematopoiesis [67]. This mechanism likely contributes to the RIs in some genes under hypoxia. However, we uncovered that the splicing factor predicted to contain the most binding sites within the *EIF2B5* intron12 locus, SRSF3, was induced at the protein level under hypoxia and exhibited increased binding at the alternatively spliced locus under hypoxia (Fig 5C and 5E). Reducing levels of SRSF3 substantially decreased expression of the 65kDa protein isoform. The full-length eIF2Bε protein was reduced as well, suggesting a major role of SRSF3 in the processing and splicing of *EIF2B5* transcripts. While there was not a significant enrichment for predicted SRSF3 binding sites for the class of 100 genes affected by RIs under hypoxia, we did identify a common feature of relatively weaker 3′ splice sites for this group of genes (Fig 6C). Previous work described that alternatively spliced loci with relatively weaker 3′ splice sites were more sensitive to changes in transcription elongation [57].

Thus, we propose that regulation of *EIF2B5* intron12 and additional introns retained under hypoxia are likely influenced by hypoxia-mediated changes in activity of RNAPII. Under hypoxia, changes in phosphorylation of the CTD of RNAPII influence expression of HIF-1α target genes by affecting the binding of cofactors and the kinetics of transcriptional activation [27]. Our observation that UV radiation was another stress that also led to an induction of 65kDa eIF2Bε strongly supports this hypothesis. UV exposure is known to induce pyrimidine dimers and other blocks to transcription elongation, which alter RNAPII transcription kinetics and subsequently impact regulation of splicing [68,69]. We posit that other stresses that have an impact on transcriptional elongation will likely impact expression of 65kDa eIF2Bε as well.

Furthermore, hypoxia-mediated changes in phosphorylation of RNAPII may explain why we observed an enrichment of splicing changes at the 3′ end of genes (i.e., ALE, TUTR, and RI categories). Several RBPs, including polyadenylation factors and splicing regulators, interact specifically with phospho-Ser2 modifications of the CTD [70]. Intriguingly, increased occupancy of phospho-Ser2 RNAPII is associated with RIs compared with constitutively spliced introns [55]. Our data detected an enrichment of phosphor-Ser2 binding under hypoxia at intron 12 in *EIF2B5*, and future work by our group will determine whether this translates to other hypoxia-induced RIs with weak 3′ splice sites as well.

The physiological relevance of these findings is underscored by the fact that *EIF2B5* intron 12 is overexpressed in tumor versus normal tissues of patients affected by 6 different cancers, including HNSC (Fig 2, S3 Fig). Interestingly, intron retention is relatively increased for nearly all solid tumors compared to normal tissue [23]. An interrogation of the TCGA database uncovered evidence of several hypoxia-induced RIs identified in this study as overexpressed in solid tumors relative to matched normal tissues (Fig 2, S3 Fig), supporting the notion that the hypoxic tumor microenvironment is a contributor to the intron retention observed in solid tumors.

There is a critical need to understand this important form of RNA processing and regulation in a cancer context. Many hypoxia-responsive isoforms, including those with RIs, have the potential to influence key biological pathways in cancer cells. For example, the hypoxia-induced RI in *TGFB1* is predicted to create a different 5′ UTR and alternate transcription start site, which would code for a 416 amino acid peptide instead of the full-length 689 amino acids. This change in the C-terminus would affect part of a FAS1 domain involved in binding integrin to regulate cell adhesion, as well as an EMI (EMILIN protein family) domain that is thought to be a protein–protein interaction domain. Additional work is needed to investigate the functional consequences of additional hypoxia-induced introns, such as those in *TGFB1* and *MARS*, predicted to create alternative protein isoforms. The identification of stress-responsive isoforms with biological functions that may differ from isoforms expressed under normal conditions will enable a deeper understanding of the link between stresses within the tumor microenvironment and regulation of RNA processing and splicing. These data will provide a new layer of information to refine prognostic hypoxia gene expression signatures and to investigate appropriate biological pathways to target hypoxic cancer cells.

## Materials and methods

### Cell lines and culture conditions

SQ20B cells, derived from human head and neck squamous cell cancer, were obtained from American Type Culture Collection (Rockville, MD). The RKO cells were a generous gift from Dr. Cho (University of Chicago). Both cell lines were maintained in Dulbecco’s Modified Eagle Medium (DMEM) media supplemented with 4.5 g/L D-Glucose, 1X L-glutamine, 10% Fetal Bovine Serum, and 1X Penicillin/Streptomycin and cultured in a 37°C humidified 5% CO_2_ atmosphere. For oxygen deprivation experiments, cells were incubated in 37°C humidified 5% CO_2_ conditions with varying concentrations of O_2_ in an INVIVO_2_ 400 chamber (Baker BioScience Solutions).

### RNA purification

RNA was isolated from cells using the Trizol reagent (ThermoFisher) and purified according to the manufacturer’s protocol. All purified RNA was subsequently treated with DNaseI digestion to remove possible DNA contaminants (Qiagen). The quality of RNA used for cDNA library preparation was verified using the RNA nano 6000 analysis chip on a BioAnalyzer 2000 series instrument (Agilent Technologies) to ensure an RNA integrity value greater than or equal to 9.

### cDNA library preparation and high-throughput sequencing

The cDNA libraries for sequencing were prepared from poly(A)+-selected mRNA, according to Illumina’s TruSeq Stranded mRNA sequencing preparation kit. Briefly, 1 μg RNA was purified for mRNA. Then mRNA material was fragmented and denatured, in preparation for first- and second-strand cDNA synthesis steps. Finally, the 3′ ends were adenylated to ligate strand-specific adapter sequences to cDNA material and amplified using PCR. Purity and size of cDNA library products were confirmed using a BioAnalyzer instrument. Library concentrations were determined via RT-qPCR using the Library Quantification Kit (KapaBiosystems). The samples were then prepared and sequenced on an Illumina HiSeq Series instrument, with 1 sample per sequencing lane to achieve > 2 x 10^8^ reads per sample.

### Analysis of RNA-sequencing data

The sequencing data were aligned using a RefSeq hg19 reference with STAR software (version 2.3.0.1), resulting in an average of 1.7 x 10^8^ uniquely aligned reads per sample. The Cufflinks software suite (version 2.1.1) was used for differential expression analysis, with standard parameters and RefSeq hg19 reference annotations. Gene ontology analyses were carried out using DAVID software [31].

The mixture of isoforms software, MISO, version 0.5.1 (February 23, 2014 release) was used for the exoncentric isoform quantification analysis. Each of the 4 hypoxia and normoxia replicates were merged into 1 file for each treatment for MISO analysis. For the exoncentric analysis, the hg19 GFF3 annotation files for each of the splicing event categories (A3SS, A5SS, AFE, ALE, MXE, RI, SE and Tandem UTR) were downloaded from http://genes.mit.edu/burgelab/miso/ as human genome (hg19) alternative events v1.0. Standard analysis parameters were used, with a filter option applied to require a minimum of 20 reads to support an event identification.

To identify regulatory elements that may affect retention of *EIF2B5* intron 12, we used AVISPA [50]. This method, based on computationally derived splicing codes, has been used previously to detect and experimentally verify novel regulators of exon splicing in a variety of experimental conditions [71–73]. Since AVISPA was built for analyzing differential splicing determinants around cassette exons, we extracted hg19 coordinates for Ensembl-defined exons 11–13 and 12–14, then analyzed the genomic regions of these 2 triplet exons using AVISPA. The loci containing triplets of exons 6–8, 7–9, 8–10, and 9–11 were also analyzed to serve as negative controls, as these exons are known to be constitutive and not exhibit intron retention. The splicing-related top motifs and regulatory features were defined by their normalized feature effect (NFE) and their relative enrichment compared to alternative and constitutive exons. Briefly, the NFE value represents the effect on splicing prediction outcome if a motif is removed in silico, normalized by the total effects observed from removing each of the top features in this way.

### Reverse transcription PCR

PCR reactions were carried out with cDNA prepared from RNA treated with DNaseI (Qiagen) to minimize contamination of DNA and in a reverse transcription reaction using oligo-dT primers to enrich for mature mRNA. Reverse transcription was carried out according to manufacturer’s protocol (Taqman RT reagents). PCR reactions were carried out in a PTC-100 Thermocycler (MJ Research, Inc.) for 40 cycles, using annealing temperatures optimal for each primer set. (Primer sequences available in Supporting Material). qPCR reactions were prepared using Power SYBR Green PCR Master Mix (Applied Biosystems) and carried out on a QuantStudio 6 Flex Real-Time PCR Instrument (Thermo Fisher Scientific). Primer sequences made available in S1 Table.

### RNAi and expression plasmid experiments

SQ20B cells were plated 24–48 h before transfection and grown to approximately 60%–70% confluency. RNAi transfections were carried out using a mixture of lipofectamine RNAi Max (ThermoFisher) diluted in OptiMEM media with siRNA to 10–50 nM, which was added to cell culture plates in complete DMEM. Cells were placed in incubator for 24 h, at which point the media was replaced. After an additional 24 h, cells were either harvested or used for subsequent experiments. For expression experiments, plasmids were purchased from Origene. Site-directed mutagenesis was used to alter the original plasmid and introduce a TAG stop codon, and confirmed by Sanger sequencing (see Fig 7A). Expression plasmids were transfected in cells plated to the same confluency as described above using a mixture of lipofectamine 2000 reagent (ThermoFisher) diluted in OptiMEM media with varying concentrations of pCMV expression plasmid added to cells in complete DMEM. The plates of cells were incubated for 4–12 h, at which point the media was washed off and replaced. Cells were harvested or used for downstream experiments 24–48 h post-transfection.

### Immunoblot analyses

Whole cell lysates were collected using a lysis buffer of 2% Triton-X, 1X Complete Mini protease inhibitor cocktail (Roche), and 1X phosphatase inhibitor cocktail 2 (Sigma) in PBS. The nuclear/cytosol fractionation reagents (BioVision) supplemented with 1X phosphatase inhibitor cocktail 2(Sigma) were used to extract cytoplasmic and nuclear extracts from the same sample. Lysates isolated from frozen tumor and normal mouse tissue were lysed in a buffer containing 1% Triton X-100, 50 mM HEPES, ph 7.4, 150 mM NaCl, 1.5 mM MgCl2, 1 mM EGTA, 100 mM NaF, 10 mM Na pyrophosphate, 1 mM Na3VO4, 10% glycerol, protease inhibitors (Roche #04693116001), and phosphatase inhibitors (Roche #04906845001). All protein concentrations were determined using DC protein assay (BioRad). Equal amounts of protein were resolved on 10% or 12% sodium dodecyl sulfate polyacrylamide gels and transferred to polyvinylidene fluoride membranes. Membranes were blocked with 5% nonfat dried milk in TBS-T (20 mM Tris, 137 mM NaCl, 0.1% Tween-20; pH 7.6) and then incubated in a 1:1,000 dilution of primary antibody in 5% milk/TBS-T followed by 1:5,000 dilution of secondary antibody in 5% milk/TBS-T. After washing, membranes were treated with ECL chemicals and exposed to autoradiograph film.

### Liquid chromatography tandem mass spectrometry

The LC-MS/MS was carried out at the Wistar core facility. Complete protocol available online: https://www.wistar.org/our-science/shared-facilities/proteomics-facility/helpful-information.

### ^35^S labeling for measurement of protein synthesis/image quantification

Following transfection experiment, SQ20B cells were incubated with methionine-/cysteine-free media for 30 m. For labeling, 1 set of experimental cell plates were incubated for 30 m with media supplemented with 0.075 mCi/ml [^35^S]-methionine/cysteine, while a second set of plates were incubated with “cold” media supplemented with nonradioactive 1X methionine/cysteine. Protein was harvested from the “cold” samples, and a protein assay was performed to obtain concentrations. These proteins were analyzed using standard immunoblot assay conditions. Proteins collected from the [^35^S]-methionine/cysteine labeled cells were resolved on SDS-PAGE. The resulting gel was then fixed using a solution of 20% methanol and 10% acetic acid for 30 m, washed with deionized water, and then incubated on a rocker at room temperature with enlightening solution (PerkinElmer) for 15–30 m. The gel was then dried for 16 h using a Bio-Rad gel-drying apparatus and then exposed to autoradiography film at -80°C for 2 h before processing.

### Polysome profiling and HPLC ATP:AMP measurements

Polysome profiling was carried out per previously described conditions [74]. The HPLC ATP:AMP protocol was performed using previously established conditions determined by our group [75].

### Chromatin immunoprecipitation

For ChIP experiments, SQ20B cells were plated 24 h before placing into hypoxia chamber or standard incubator. Six 10-cm plates at 70% confluency were grown in normoxic or hypoxic (0.5% O_2_) conditions for 16 h, at which time cells were crosslinked for 10 m at room temperature using 1% formaldehyde in minimum essential medium. Crosslinking was stopped and cells were washed and lysed according to manufacturer’s protocol (Active Motif ChIP kit #53035). Shearing conditions were carried out for six 20-s pulses at 25% power, with 30 s rest in between pulses on ice. Immunoprecipitations were carried out according to protocol, with 40 ug chromatin and 3 ug antibody for RNAPII (Active Motif #39097, mouse IgG #53010) and 60 ug chromatin with 3 ug (rabbit IgG Santa Cruz #sc-2027X) or 30 ug P-Ser2 RNAPII (Abcam #5095). For the resulting qPCR reactions, primers specific to *EIF2B5* intron 10 and intron 12 were used (S1 Table), and control primers were purchased from Active Motif (GAPDH-2 for RNAPII control #71006, GAPDH-1 for P-Ser2 RNAPII control #71004, and Negative-1 for a 78-bp intergenic region of chromosome 12 as a negative control #71001).

### RNA immunoprecipitation

SQ20B cells were grown in 2 x150-cm tissue culture plates for each condition (normoxia and a 0.5% hypoxia time point). Antibody against SRSF3 (SRp20) was purchased from MBL, Inc. (#RN080PW). Rabbit IgG was provided by the RIP kit for use as a control (RIP Assay kit, MBL, Inc. # RN1001). Protein A Sepharose CL-4B (GE Healthcare) were prepared fresh for the beginning of the experiment in a slurry of 75% beads in 25% 50 mM Tris pH 7.5. Beads were prewashed in PBS before adding 15 μg of antibody and then incubated at 4 degrees Celsius with rotation for 4 h. At this time, beads were prewashed before lysing cells and adding the lysates to the Protein A beads to preclear for 1 h at 4 degrees with rotation. Input samples were collected at this time before adding the precleared lysate to the antibody-bound beads. Samples were incubated for 3 h at 4 degrees with rotation. After wash steps, samples were aliquoted for quality control analysis of the protein. RNA was isolated according to the manufacturer’s protocol and analyzed using the Nanodrop1000 UV spectrometer. Equal amounts of RNA were then processed into cDNA and used for subsequent qPCR analysis. Input RNA was used as a standard to calculate the quantity for each primer. Expression data are reported as the relative enrichment of SRSF3 immunoprecipitation over the control Rabbit IgG immunoprecipitation.

### Statistical analyses

All statistical analyses and drawings were done in R (version 3.2.5) (http://www.r-project.org/), and the statistical significance was defined as a *P* value <0.05.

For gene expression levels of the regions of interest, we downloaded RPKM data from TANRIC (http://ibl.mdanderson.org/tanric/_design/basic/index.html) [76]. The expression data is log2 transformed.

We restricted ourselves to the 8 types of cancers with available data for at least 30 normal samples. These are breast invasive carcinoma (BRCA), HNSC, KIRC, kidney renal papillary cell carcinoma (KIRP), liver hepatocellular carcinoma (LIHC), lung adenocarcinoma (LUAD), prostate adenocarcinoma (PRAD), and thyroid carcinoma (THCA). We downloaded patient clinical information for the patients of these cohorts from cbioPortal (http://www.cbioportal.org/).

To be able to determine the expression difference for the regions of interest between normal and tumor tissue, respectively, among normal and tumor tissue separated according to the cancer stage, we first employed a Shapiro-Wilk test to verify if the data follows a normal distribution. Accordingly, *t* test, respectively ANOVA test (depending on the number of groups considered), or the nonparametric test Mann-Whitney-Wilcoxon, respectively Kruskal-Wallis test, was applied to assess the relationship between mRNA expression and tissue type.

A box-and-whisker plot (Box plot represents first [lower bound] and third [upper bound] quartiles, whiskers represent 1.5 times the interquartile range) was used to visualize the data.

## Supporting information

S1 Fig RT-qPCR validation of hypoxia-induced changes in gene and isoform expression detected by RNAseq. For each set of graphs, RNAseq data shown to the left and corresponding RT-qPCR shown at right (average of 3, independent experiments +/- S.E.M.) for SQ20B cells in normoxia or 0.5% O_2_ for 16 h. (A) Expression of select HIF-1α target genes. (B) Induced isoforms, with isoform 1, 2, or 3 designated with number following gene symbol. (C) Repressed isoforms, with isoform 1, 2, or 3 labeled. Data available in Supporting Information S1 Data file. Note: In the RNA-Seq graph for panel (C), NEK6_2 and FAM86C_3 isoforms are represented with approximate Fold-change values of “-5” for visualization but were undetected (FPKM = 0) in hypoxia samples by RNA-Seq.
(TIF)

S2 Fig PCR validation of hypoxia-induced retained introns in SQ20B cells identified by RNAseq. Quantification of 3 independently conducted PCR experiments shown PCR for hypoxia-induced retained introns in 4 genes, including A: *ANKZF1*, B: *EIF2B5*, C: *MARS* and D: *TGFB1*. (ImageJ software used for quantification). *P*-values reported for statistically significant differences as determined by Student *t* test. Data used to generate this figure available in Supporting Information S1 Data file.
(TIF)

S3 Fig Expression analysis of intron expression in tumors versus matched normal tissues for several solid cancer datasets from The Cancer Genome Atlas (TCGA). (A-E) Each panel represents a different cancer dataset. Abbreviations: HNSC: head and neck squamous cell carcinoma, LIHC: liver hepatocellular carcinoma, LUAD: lung adenocarcinoma, KIRC: kidney renal clear cell carcinoma, KIRP: kidney renal papillary cell carcinoma, PRAD: prostate adenocarcinoma. Data used to generate this figure available in Supporting Information S1 Data file.
(TIF)

S4 Fig Additional *EIF2B5* splice junction analysis. Splice graph of 3′ end of *EIF2B5* displaying splice junction reads for all normoxia and hypoxia samples, with arrow signifying intron 12 (created using MAJIQ/VOILA). RNAseq expression data are reported as mean, normalized read counts at each junction for all 4 normoxia and 4 hypoxia biological replicates. Normalized read count displayed over each junction to highlight decrease in reads at junctions following intron 12 as opposed to before intron 12.
(TIF)

S5 Fig Additional validation of a stress-induced 65kDa protein isoform of eIF2Bε. (A) Additional hypoxia time-course of SQ20B cells treated with siRNA against *EIF2B5* (see Fig 3C for siRNA experiment in 0.2% O_2_ conditions). Abbreviation: NT, non-targeting siRNA. (B) Immunoblot of lysates from SQ20B cells treated with thapsigargin to induce endoplasmic reticulum stress. Control cells were treated with DMSO. (C) Cells expressing control empty vector (EV) pCVM6.AC plasmid or plasmid expressing wild-type (WT) full-length eIF2Bε with a C-terminal Flag-tag sequence were subject to UV exposure and then collected 4 h later to test for evidence of cleavage or degradation products. Only product sizes consistent with tagged expression of full-length eIF2Bε were evident on the immunoblot incubated with α–Flag antibody. Expect to see appearance of 18.2 kDa band here if cleaved, but it appears at expected ~85kDa (size of full-length eIF2Bε plus the Flag-tag) as evidence that there is no cleavage of the protein under UV stress. (D) Analysis of siRNA knock-down efficiency of UPF1 and resulting impact of eIF2Bε protein expression in normoxic SQ20B cells.
(TIF)

S6 Fig Predicted binding and functional impact of select factors on regulation of splicing of *EIF2B5*. (A) Binding map within *EIF2B5* locus which contains intron 12 (highlighted in blue) with splicing factors identified as hypoxia-responsive shown. Map generated using custom UCSC Genome Browser track produced from RBPmap. (B) Immunoblot of SQ20B nuclear lysates to show expression of CELF5 under hypoxia. (C)Additional immunoblot of siRNA experiment shown in Fig 4D, but in normoxic conditions. (D)Knock-down efficiency of CELF5 siRNA shown by immunoblot.
(TIF)

S7 Fig Hypoxia-induced phosphorylation of RNA polymerase II. (A) Immunoblot of nuclear lysates collected from hypoxia time-course of SQ20B cells. (B) Immunoblot of nuclear lysates collected from hypoxia time-course of SQ20B cells. (C) Quantification of 3 independently conducted experiments (including replicate from Fig 6) carried out using ImageJ software. *P*-value reported for statistically significant difference as determined by Student *t* test. Data used to generate this figure available in S1 Data.
(TIF)

S8 Fig Additional polyribosome profile analyses in SQ20B cells after 48 h expression of eIF2Bε 65kDa isoform. (A) SQ20B Cells expressing the 65kDa isoform of eIF2Bε for 48 h display an overall reduction in the polyribosome profile compared to cells expressing empty vector (EV). (B) HPLC analysis of ATP:AMP levels in SQ20B cells expressing pCMV6 control plasmid, or plasmid expressing full-length or 65kDa eIF2Bε. Data are shown as average of 2 biological replicates. (C) Clonogenic assay of SQ20B cells expressing control plasmid or plasmid containing 65kDa eIF2Bε in cells grown in normoxic or 16 h hypoxic conditions. Surviving fraction for 3 biological replicates reported in Fig 7. (D) Clonogenic assay of SQ20B cells expressing control plasmid or plasmid containing 65kDa eIF2Bε in cells grown in normoxic or 24 h hypoxic conditions. Survival fraction for 3 biological replicates reported in Fig 7.
(TIF)

S1 Data Additional data used in the generation of the figures in the manuscript and Supporting Information. Includes raw data for Figs 1, 2, 3, 5, 6, and 7 and S1, S2, S3, S7, and S8 Figs.
(XLSX)

S1 Table List of primer sequences and additional information for reagents used in the experiments included in this manuscript. (DOCX)

## Abbreviations

ALE: alternate last exon
AMP: Adenosine monophosphate
ATP: Adenosine triphosphate
CELF: CUGBP Elav-Like Family
CTD: C-terminal domain
EMI: EMILIN protein family
ER: endoplasmic reticulum
FDR: false discovery rate
FPKM: Fragments Per Kilobase of transcript per Million mapped reads
GDP: guanosine diphosphate
GEF: guanine exchange factor
GTP: guanosine triphosphate
HIF: Hypoxia-Inducible transcription Factor
HNSC: head and neck squamous cell carcinoma
HRE: hypoxia-responsive element
KIRC: kidney renal clear cell carcinoma
LC-MS/MS: liquid chromatography tandem mass spectrometry
NMD: nonsense-mediated decay
PTC: premature termination codon
qPCR: quantitative PCR
RBP: RNA binding protein
RI: retained intron
RNAPII: RNA polymerase II
siRNA: small interfering RNA
TCGA: The Cancer Genome Atlas
TUTR: tandem 3′ UTRs
UPR: unfolded protein response
UV: ultraviolet
