# Supplementary figures and images for: Transcriptome analysis of hypoxic cancer cells uncovers intron retention in EIF2B5 as a mechanism to inhibit translation
Source: PLoS Biol. 2017 Sep 29;15(9):e2002623. doi: 10.1371/journal.pbio.2002623 (PMC5636171; doi:10.1371/journal.pbio.2002623)

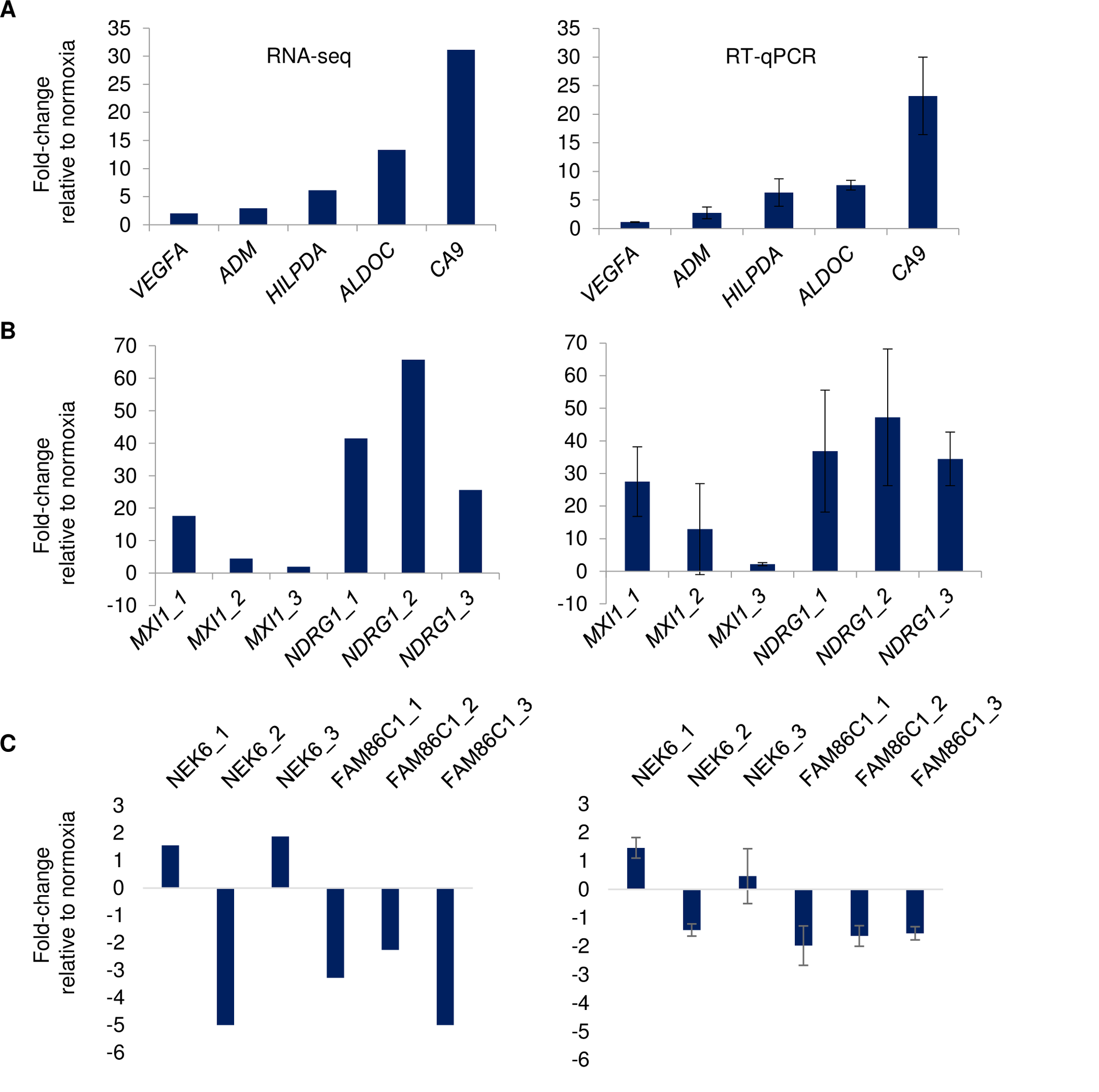

Supplement: S1 Fig — For each set of graphs, RNAseq data shown to the left and corresponding RT-qPCR shown at right (average of 3, independent experiments +/- S.E.M.) for SQ20B cells in normoxia or 0.5% O2 for 16 h. (A) Expression of select HIF-1α target genes. (B) Induced isoforms, with isoform 1, 2, or 3 designated with number following gene symbol. (C) Repressed isoforms, with isoform 1, 2, or 3 labeled. Data available in Supporting Information S1 Data file. Note: In the RNA-Seq graph for panel (C), NEK6_2 and FAM86C_3 isoforms are represented with approximate Fold-change values of “-5” for visualization but were undetected (FPKM = 0) in hypoxia samples by RNA-Seq. (TIF) [file pbio.2002623.s001.tif]

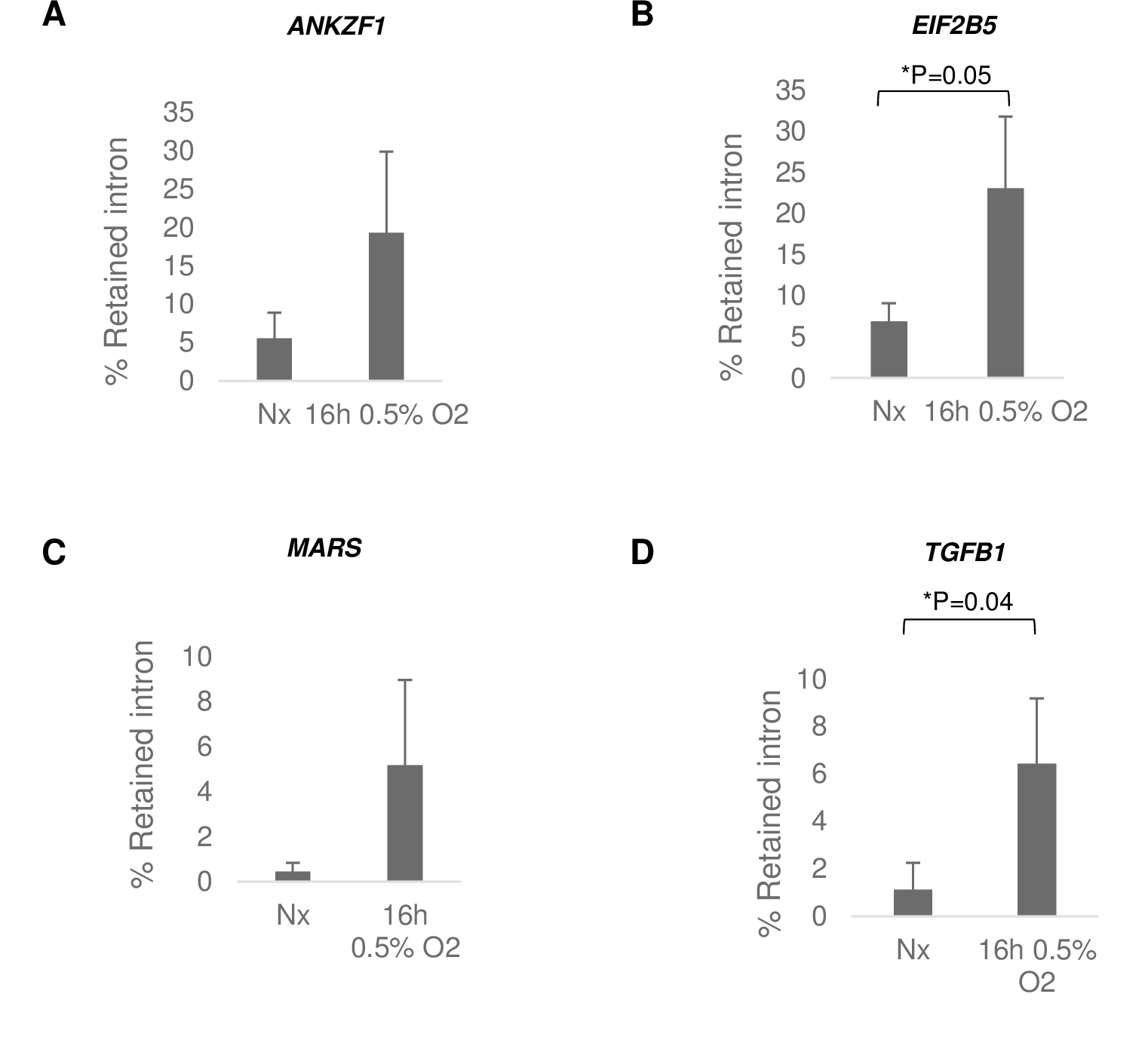

Supplement: S2 Fig — Quantification of 3 independently conducted PCR experiments shown PCR for hypoxia-induced retained introns in 4 genes, including A: ANKZF1, B: EIF2B5, C: MARS and D: TGFB1. (ImageJ software used for quantification). P-values reported for statistically significant differences as determined by Student t test. Data used to generate this figure available in Supporting Information S1 Data file. (TIF) [file pbio.2002623.s002.tif]

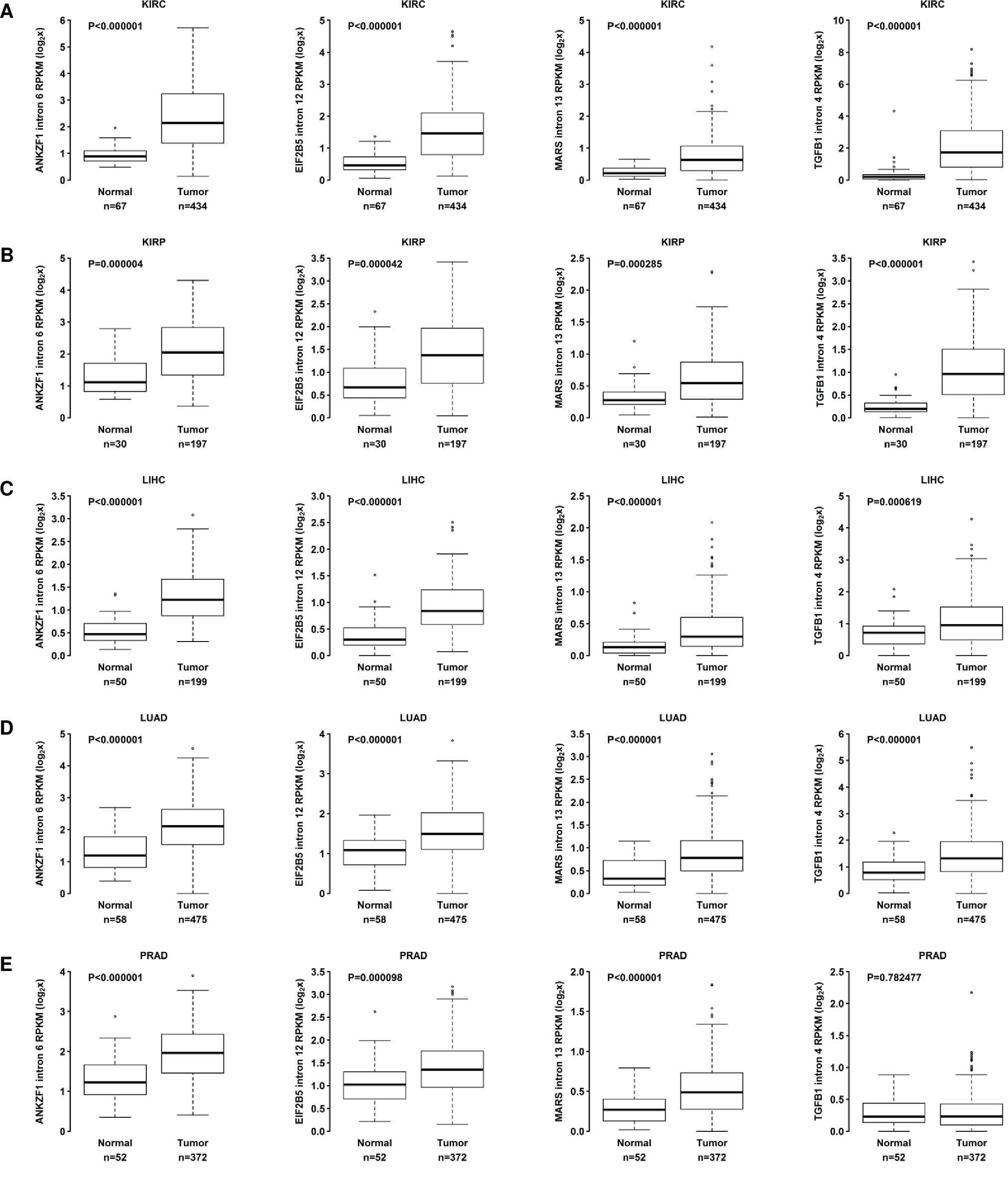

Supplement: S3 Fig — (A-E) Each panel represents a different cancer dataset. Abbreviations: HNSC: head and neck squamous cell carcinoma, LIHC: liver hepatocellular carcinoma, LUAD: lung adenocarcinoma, KIRC: kidney renal clear cell carcinoma, KIRP: kidney renal papillary cell carcinoma, PRAD: prostate adenocarcinoma. Data used to generate this figure available in Supporting Information S1 Data file. (TIF) [file pbio.2002623.s003.tif]

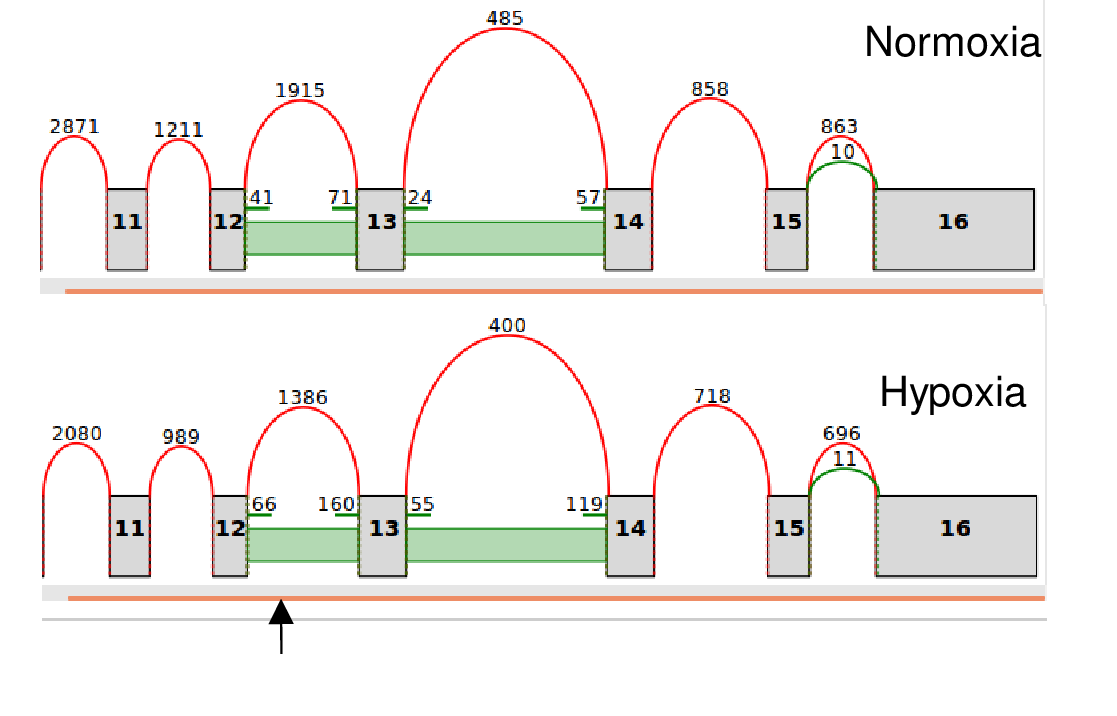

Supplement: S4 Fig — Splice graph of 3′ end of EIF2B5 displaying splice junction reads for all normoxia and hypoxia samples, with arrow signifying intron 12 (created using MAJIQ/VOILA). RNAseq expression data are reported as mean, normalized read counts at each junction for all 4 normoxia and 4 hypoxia biological replicates. Normalized read count displayed over each junction to highlight decrease in reads at junctions following intron 12 as opposed to before intron 12. (TIF) [file pbio.2002623.s004.tif]

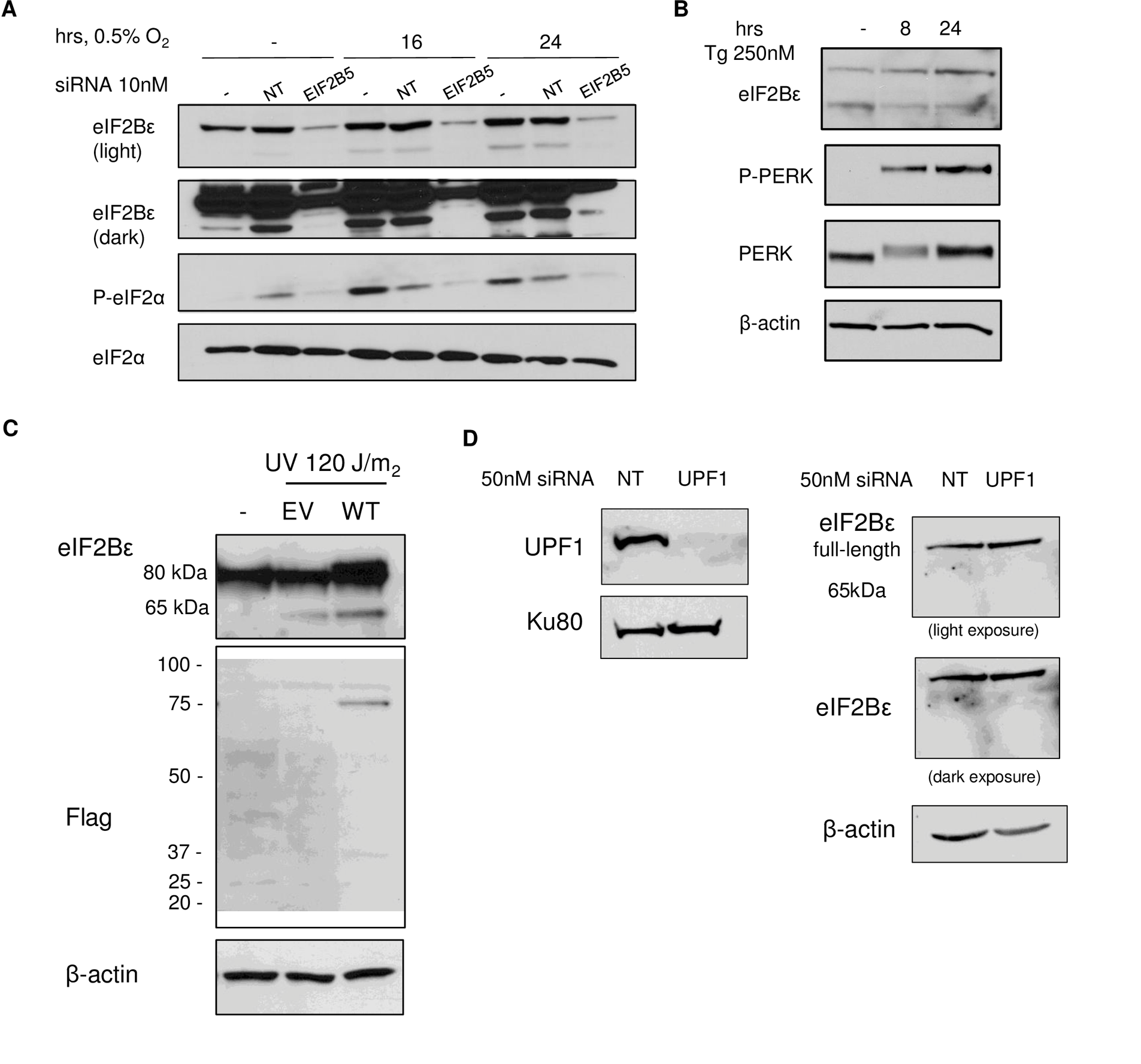

Supplement: S5 Fig — (A) Additional hypoxia time-course of SQ20B cells treated with siRNA against EIF2B5 (see Fig 3C for siRNA experiment in 0.2% O2 conditions). Abbreviation: NT, non-targeting siRNA. (B) Immunoblot of lysates from SQ20B cells treated with thapsigargin to induce endoplasmic reticulum stress. Control cells were treated with DMSO. (C) Cells expressing control empty vector (EV) pCVM6.AC plasmid or plasmid expressing wild-type (WT) full-length eIF2Bε with a C-terminal Flag-tag sequence were subject to UV exposure and then collected 4 h later to test for evidence of cleavage or degradation products. Only product sizes consistent with tagged expression of full-length eIF2Bε were evident on the immunoblot incubated with α–Flag antibody. Expect to see appearance of 18.2 kDa band here if cleaved, but it appears at expected ~85kDa (size of full-length eIF2Bε plus the Flag-tag) as evidence that there is no cleavage of the protein under UV stress. (D) Analysis of siRNA knock-down efficiency of UPF1 and resulting impact of eIF2Bε protein expression in normoxic SQ20B cells. (TIF) [file pbio.2002623.s005.tif]

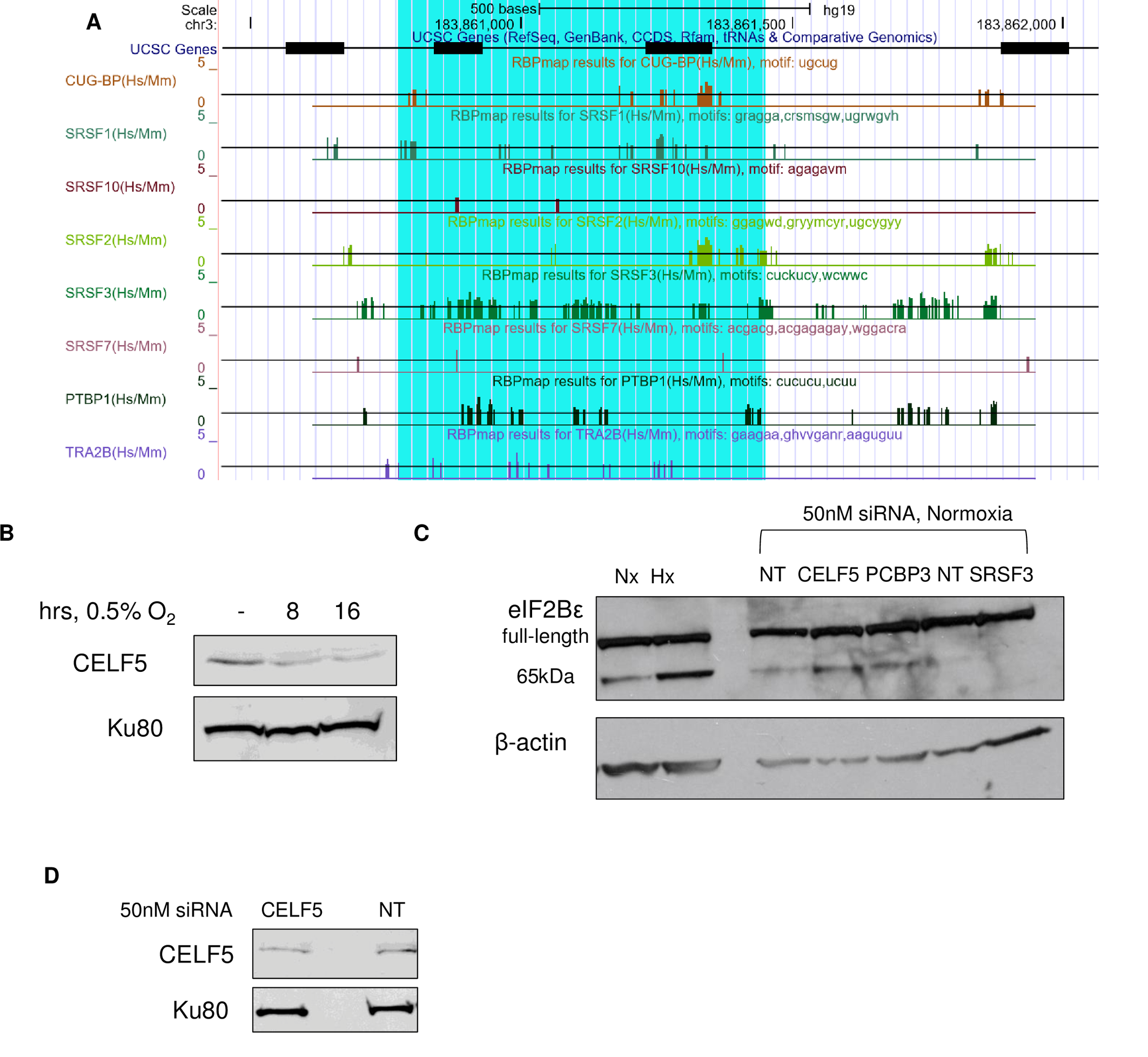

Supplement: S6 Fig — (A) Binding map within EIF2B5 locus which contains intron 12 (highlighted in blue) with splicing factors identified as hypoxia-responsive shown. Map generated using custom UCSC Genome Browser track produced from RBPmap. (B) Immunoblot of SQ20B nuclear lysates to show expression of CELF5 under hypoxia. (C)Additional immunoblot of siRNA experiment shown in Fig 4D, but in normoxic conditions. (D)Knock-down efficiency of CELF5 siRNA shown by immunoblot. (TIF) [file pbio.2002623.s006.tif]

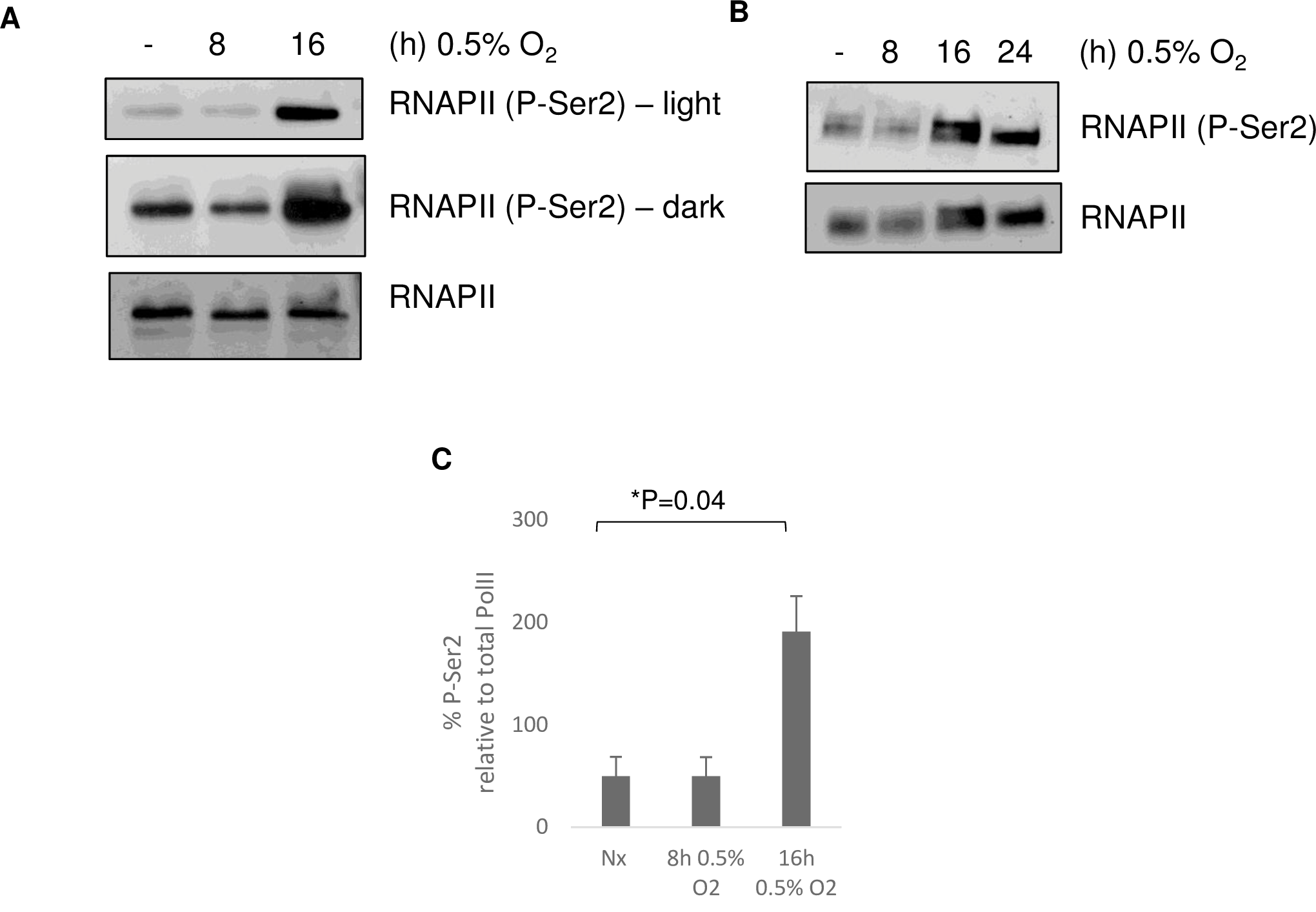

Supplement: S7 Fig — (A) Immunoblot of nuclear lysates collected from hypoxia time-course of SQ20B cells. (B) Immunoblot of nuclear lysates collected from hypoxia time-course of SQ20B cells. (C) Quantification of 3 independently conducted experiments (including replicate from Fig 6) carried out using ImageJ software. P-value reported for statistically significant difference as determined by Student t test. Data used to generate this figure available in S1 Data. (TIF) [file pbio.2002623.s007.tif]

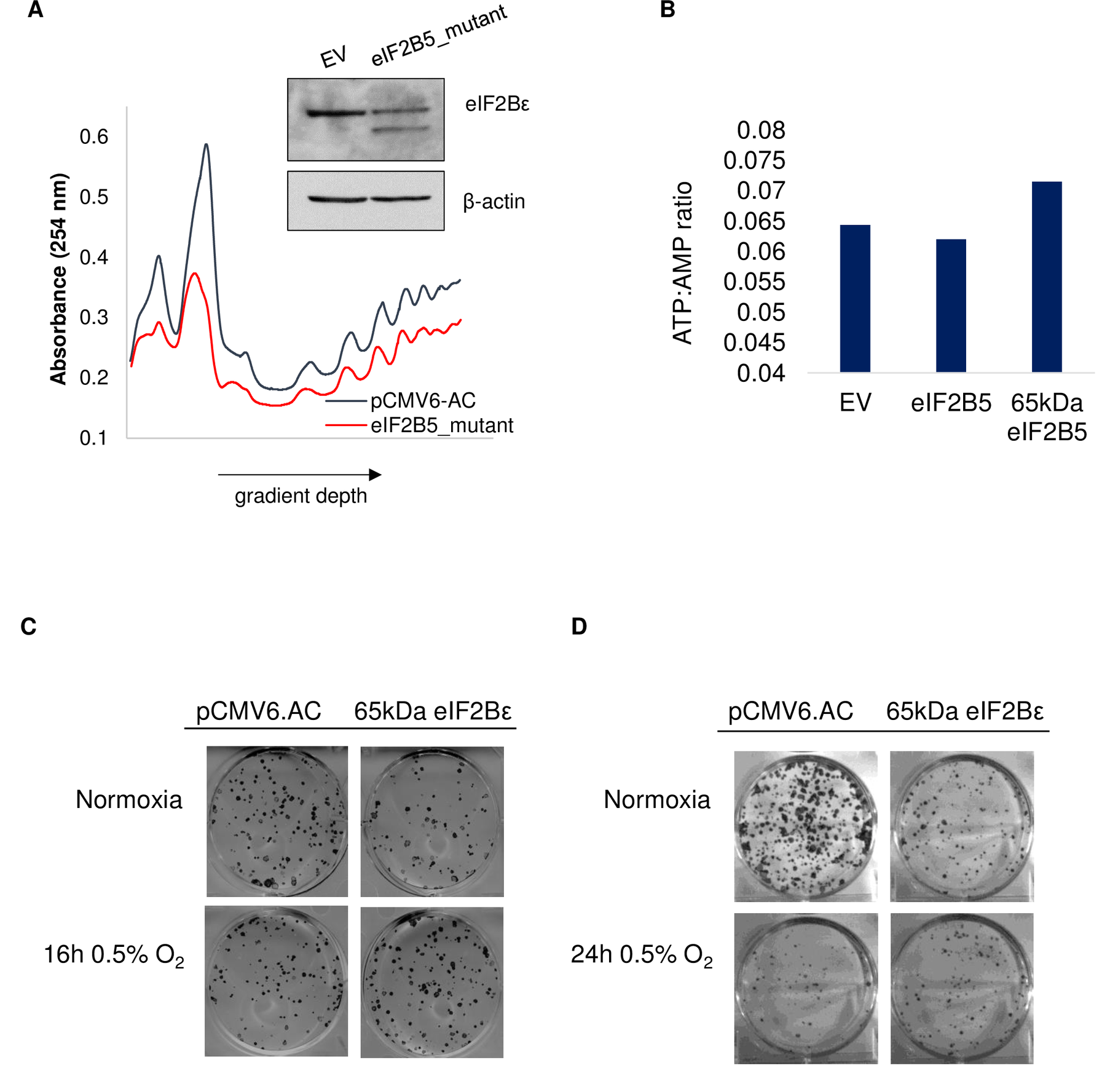

Supplement: S8 Fig — (A) SQ20B Cells expressing the 65kDa isoform of eIF2Bε for 48 h display an overall reduction in the polyribosome profile compared to cells expressing empty vector (EV). (B) HPLC analysis of ATP:AMP levels in SQ20B cells expressing pCMV6 control plasmid, or plasmid expressing full-length or 65kDa eIF2Bε. Data are shown as average of 2 biological replicates. (C) Clonogenic assay of SQ20B cells expressing control plasmid or plasmid containing 65kDa eIF2Bε in cells grown in normoxic or 16 h hypoxic conditions. Surviving fraction for 3 biological replicates reported in Fig 7. (D) Clonogenic assay of SQ20B cells expressing control plasmid or plasmid containing 65kDa eIF2Bε in cells grown in normoxic or 24 h hypoxic conditions. Survival fraction for 3 biological replicates reported in Fig 7. (TIF) [file pbio.2002623.s008.tif]
