# Supplementary material for: Transcriptome analysis of hypoxic cancer cells uncovers intron retention in EIF2B5 as a mechanism to inhibit translation
Source: PLoS Biol. 2017 Sep 29;15(9):e2002623. doi: 10.1371/journal.pbio.2002623 (PMC5636171; doi:10.1371/journal.pbio.2002623)
Supplement: S1 Table — (DOCX) [file pbio.2002623.s010.docx]

Table S1: Reagents used in this manuscript.

| Induced isoforms | Target mRNA | Sequence |
| --- | --- | --- |
|  |  |  |
| Induced isoforms | Target mRNA | Sequence |
| NDRG1_F1 | NM_001135242 | GACTAGGCAGGTGACAGC |
| NDRG1_F2 | NM_006096,NM_001258433 | TCGCGTTAGGCAGGTGA |
| NDRG1_R1 | NM_001135242,NM_006096, NM_001258433 | ACTCCACCAAAGGCTTCA |
| NDRG1_F3 | NM_001258432 | TCGCGTTAGGAGCAGGA |
| NDRG1_R2 | NM_001258432 | TGGTAGGTGAGGATGACAGG |
| MXI1_F1 | NM_130439 | GAAAACAAAAAGTCTGAACATG |
| MXI1_F2 | NM_001008541 | CTAAGTCAACTTCATAGTGT |
| MXI1_F3 | NM_005962 | GGGAGCGAGAGTGTGAACAT |
| MXI1_R | common to all | TTGCTGCTCCCGCTGCT |
|  |  |  |
| Repressed isoforms | Target mRNA | Sequence |
|  |  |  |
| FAM86C1_F1 | NM_018172 | GGAGCTATTTGCTGACGTGC |
| FAM86C1_R1 | NM_018172 | TGTAGAATTGAGGAGCCCGC |
| FAM86C1_F2 | NM_001099653 | GTCAGTCAAGTATGCCCGGTG |
| FAM86C1_R2 | NM_001099653 | GGCCATGTAGAATTGAGGAGC |
| FAM86C1_F3 | NM_152563 | ATTTTGCAGAAGACGTGCTGT |
| FAM86C1_R3 | NM_152563 | GACGGTAAGGGCCATGTAGAAT |
| NEK6_F1 | NM_001166171 | CTCAGGAGCCACTTCAAAAGG |
| NEK6_R1 | NM_001166171 | CCTCCCAGCAAACTTCTCTCC |
| NEK6_F2 | NM_001166168 | GTGGCGTTGTTGGGTTCGT |
| NEK6_R2 | NM_001166168 | TGTGGCAGAGGTTGTTGGAA |
| NEK6_F3 | NM_001166170 | CACTTCAAAAGTTCGTGCCCTC |
| NEK6_R3 | NM_001166170 | ATGCCTCTGTGGGTCAGGAG |
|  |  |  |
| Other |  |  |
| EIF2B5_F | total gene | CATCAAAGTGTTCCAGAA |
| EIF2B5_R | total gene | TATAGGCATACTTGAGAGAG |
| int12_f | EIF2B5_intron12 | CAAATGGAAAGACCAGTAGTATT |
| int12_r | EIF2B5_intron12 | AGAACTATGACTTTGATAGACATTG |
| Int10_f | EIF2B5_intron10 | CTCCTGAATCGGAATATTTTGAAGG |
| Int10_r | EIF2B5_intron10 | CTGAGAAGCCATAATGGGTGA |
| EIF2B5ex12_RIP_F | EIF2B5 exon 12 | TGAAGTTTTAGGAACACTACAG |
| EIF2B5ex12_RIP_R | EIF2B5 exon 12 | GAGAGAGTTGATTTCCAGG |
| EIF2B5ex13_RIP_F | EIF2B5 exon 13 | CATAAGTCTAAAGGAGGTGATG |
| EIF2B5ex13_RIP_R | EIF2B5 exon 13 | CACAGTAGCGGCTTGAGTCAAG |
|  |  |  |
| Control genes |  |  |
| s14_f | total gene | GGCAGACCGAGATGAATCCTC |
| s14_r | total gene | CAGGTCCAGGGGTCTTGGTCC |
| VEGFA_F | total gene | GCTCGGTGCTGGAATTGGAT |
| VEGFA_R | total gene | GCCCGATTCAAGTGGGGAAT |
| ADM_F | total gene | AATGAATGCTGAACCCCCG |
| ADM_R | total gene | TGACACGCCGTGAGAAATCA |
| HILPDA_F | total gene | AACCGACTTTCCTCCGGACT |
| HILPDA_R | total gene | GGCTGAAAGGACCCTACTCC |
| ALDOC_F | total gene | TGCCTCTAGCTGGGACTGAT |
| ALDOC_R | total gene | CACTTGGCAAAGTCAGCACC |
|  |  |  |
|  |  |  |
| PCR primer | Target | Sequence |
| EIF2B5_F_INT12 | Retained intron | ATGACATCAAAGTGTTCCAGA |
| EIF2B5_R_INT12 | Retained intron | TTAGCAGAGGAAGCAGCA |
| ANKZF1_F | Retained intron | ACAATACTGTTGCGTGCTCC |
| ANKZF1_R | Retained intron | CGTAGGCATCTGGATTCTTC |
| MARS_F | Retained intron | TTGAAGACAAGGTATTCTATGTCTGGT |
| MARS_R | Retained intron | AGCAATGAGGTGGCTGACCAA |
| TGFB1_F | Retained intron | ACTCTCAAACCTTTACGAGACCCT |
| TGFB1_R | Retained intron | GTTCACAGTTACAATCCCATTAGGATA |
|  |  |  |
|  |  |  |
| siGENOME SMARTpool | Target: EIF2B5 | sequence |
| siRNA D-012625-18 | gene pool | CGACCAUUUGGAAGCGUUA |
| siRNA D-012625-17 | gene pool | UCUCAAUGUGGUUCGAAUA |
| siRNA D-012625-03 | gene pool | GCACGUAACAGCUAAGGAA |
| siRNA D-012625-01 | gene pool | GCAUGAAGCUCUUGGUAUU |
|  |  |  |
| custom designed oligos | EIF2B5_int12 | sequence |
| custom1 | intron12 | GGAAAGACCAGUAGUAUUUUU |
| custom2 | intron12 | GGUAGAGGCUUUCUCGUAAUU |
|  |  |  |
| Antibody | Dilution | Source |
| Phospho-eIF2α (ser51) (119A11) | 1/1000 | Cell Signaling |
| Total eIF2α | 1/1000 | Cell Signaling |
| eIF2Bε | 1/1000 | Bethyl |
| β-actin | 1/1000 | Sigma |
| Phospho-PERK (Thr980) (16F8) | 1/1000 | Cell Signaling |
| PERK | 1/1000 | Cell Signaling |
| SRSF3 | 1/1000 | Abcam |
| CUG-BP | 1/1000 | Abcam |
